# Meta‐Analysis: Prevalence of Non‐ *Helicobacter pylori* , Non‐NSAID Peptic Ulcer Disease

**DOI:** 10.1111/apt.70906

**Published:** 2026-08-10

**Authors:** Jun Watanabe, Hiromi Sekiguchi, Takeshi Kanno, Keigo Misawa, Satoshi Sato, Yuhong Yuan, Paul Moayyedi, Tomonori Yano, Atsushi Masamune, Tomoari Kamada

**Affiliations:** ^1^ Department of Surgery, Division of Gastroenterological, General and Transplant Surgery Jichi Medical University Shimotsuke Tochigi Japan; ^2^ Division of Community and Family Medicine Jichi Medical University Shimotsuke Tochigi Japan; ^3^ Department of Medicine, Division of Gastroenterology and Farncombe Family Digestive Health Research Institute McMaster University Hamilton Ontario Canada; ^4^ Department of Medicine, Division of Gastroenterology Jichi Medical University Shimotsuke Tochigi Japan; ^5^ R & D Division of Career Education for Medical Professionals, Medical Education Center Jichi Medical University Shimotsuke Tochigi Japan; ^6^ Division of Gastroenterology Tohoku University Graduate School of Medicine Sendai Miyagi Japan; ^7^ Department of Health Care Medicine Kawasaki Medical School General Medical Center Kurashiki Okayama Japan

**Keywords:** anti‐inflammatory agents, gastrointestinal haemorrhage, *Helicobacter pylori*, idiopathic peptic ulcers, nonsteroidal, peptic ulcer disease

## Abstract

**Background:**

Non‐ *Helicobacter pylori* ( *H. pylori* ), non‐nonsteroidal anti‐inflammatory drug (NSAID) peptic ulcer disease (PUD) is increasingly recognized as a distinct subgroup, but its proportion across clinical presentations remains unclear.

**Aim:**

We aimed to estimate this proportion.

**Methods:**

We searched MEDLINE, Embase and CENTRAL from their inception to March 2026 for observational studies. The primary outcome was the proportion of non‐ *H. pylori* , non‐NSAID PUD among adults with overall, bleeding and perforated PUD. Random‐effects meta‐analysis of logit‐transformed proportions was performed using restricted maximum likelihood. Meta‐regression assessed associations with study year, country‐level *H. pylori* prevalence and region. Protocol registered in PROSPERO (CRD420251174202).

**Results:**

Overall, 92 studies (60,816 patients) from 32 countries were included. The pooled proportions of non‐ *H. pylori* , non‐NSAID PUD cases were 13.0% (95% confidence interval [CI], 10.4–16.2) in overall PUD, 11.3% (95% CI, 8.8–14.4) in bleeding PUD, and 22.0% (95% CI, 13.1%–34.7%) in perforated PUD. In univariable meta‐regression, a more recent study year was associated with a higher proportion of bleeding PUD (*β* = 0.076; 95% CI, 0.031–0.120). This association remained statistically significant after adjustment for country‐level *H. pylori* prevalence (*β* = 0.070; 95% CI, 0.026–0.113). Lower *H. pylori* prevalence was also associated with a higher proportion after further adjustment for region (*β* = −0.025; 95% CI, −0.046 to −0.005).

**Conclusion:**

Non‐ *H. pylori* , non‐NSAID PUD represents a non‐negligible but heterogeneous component of PUD. In bleeding PUD, exploratory ecological analyses suggest a temporal increase, potentially reflecting evolving etiologic patterns beyond *H. pylori* infection and NSAID exposure.

## Graphical abstract

A meta‐analysis of 92 studies including 60,816 patients found that non‐ *H. pylori* , non‐NSAID represent a non‐negligible but heterogeneous component of peptic ulcer disease. Among bleeding ulcers, this proportion increased over time, potentially reflecting evolving etiologic patterns beyond *H. pylori* infection and NSAID exposure.

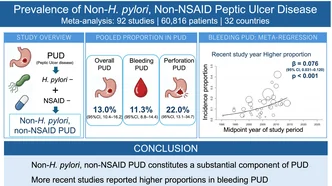

**Abbreviations**
CI: confidence interval

*H. pylori*

:
*Helicobacter pylori*

PUD: peptic ulcer disease

## 1 Introduction

Peptic ulcer disease (PUD) remains a major cause of upper gastrointestinal bleeding and associated mortality [1, 2, 3]. It accounts for approximately 60%–70% of cases requiring emergency endoscopy for upper gastrointestinal bleeding, with a 30‐day mortality of about 10% [4, 5]. *Helicobacter pylori* infection and nonsteroidal anti‐inflammatory drug (NSAID) use are the two primary established causes of PUD [6, 7, 8]. However, some ulcers occur in the absence of both factors and are classified as non‐ *H. pylori* , non‐NSAID PUD [1, 9]. This subgroup has been associated with severe comorbidities, lower healing rates, and higher risks of recurrence and rebleeding compared with other forms of PUD [9, 10, 11].

Defining the proportion of non‐ *H. pylori* , non‐NSAID PUD may clarify evolving etiologic patterns and help identify high‐risk populations [12]. However, its global epidemiology and temporal trends remain poorly characterized. Therefore, we conducted a systematic review and meta‐analysis to estimate the proportion of non‐ *H. pylori* , non‐NSAID PUD among adults with overall, bleeding, and perforated PUD and to examine temporal trends. Understanding these patterns may inform diagnostic strategies, guide preventive interventions and support healthcare resource allocation.

## 2 Methods

### 2.1 Protocol

We followed the Preferred Reporting Items For Systematic Reviews and Meta‐Analyses 2020 (PRISMA‐2020) guidelines [13]. The protocol was registered in PROSPERO (CRD420251174202) (Appendices S1–S3).

### 2.2 Study Selection

We included observational (prevalence) studies reporting the proportion of non‐ *H. pylori* , non‐NSAID PUD among adults with overall, bleeding, or perforated PUD. Randomized controlled trials, case reports, case series, reviews and letters were excluded. Adult patients (aged ≥ 18 years) diagnosed with gastric and/or duodenal ulcers were eligible. Studies that did not report the proportion of non‐ *H. pylori* , non‐NSAID PUD or lacked sufficient data to calculate it were excluded. Studies including malignant or post‐operative ulcers were also excluded.

### 2.3 Types of Outcome Measures

The primary outcome was the proportion of non‐ *H. pylori* , non‐NSAID PUD among adults with overall, bleeding, or perforated PUD.

### 2.4 Electronic Searches

We searched MEDLINE, Embase and Cochrane CENTRAL from 1990 to March 21, 2026, without language or country restrictions. We conducted an initial search on September 26, 2025, and updated the search on March 21, 2026. Detailed search strategies are provided in Appendix S4. We also screened the reference lists of international guidelines [1, 2, 3] and hand‐searched the references and citations of the studies identified.

### 2.5 Data Collection

Two authors (JW and HS) independently screened titles and abstracts, assessed full texts, extracted study and patient characteristics, and evaluated risk of bias using the Joanna Briggs Institute Prevalence Critical Appraisal Tool [14]. Discrepancies were resolved through discussion with a third reviewer (TK). For subgroup analyses, studies were classified as low, moderate, or high risk of bias according to predefined decision rules, which were not formally validated by Joanna Briggs Institute (details in Appendices S1–S12).

### 2.6 Statistical Analysis

We performed a meta‐analysis of logit‐transformed proportions using a random‐effects model with restricted maximum‐likelihood estimation. Pooled estimates and 95% confidence intervals (CIs) were back‐transformed and reported as percentages. For random‐effects meta‐analyses, 95% prediction intervals (PIs) were also calculated and back‐transformed to indicate the expected range of true proportions in comparable future settings. Statistical heterogeneity was assessed using the *I* ^2^ statistic. However, high *I* ^2^ values are common in meta‐analyses of proportions; therefore, results were interpreted alongside subgroup analyses and meta‐regression rather than using fixed *I* ^2^ thresholds [15]. Funnel plots and Egger's test were not performed, as their assumptions and validity have not been established for proportional outcomes.

We also estimated the pooled proportions of *H. pylori* ‐associated ulcers, NSAID‐associated ulcers and ulcers associated with both *H. pylori* infection and NSAID use among overall PUD cases and separately among bleeding PUD cases. Potential sources of heterogeneity, prespecified a priori, were evaluated through subgroup analyses by study period duration (≤ 1, > 1 to ≤ 3, > 3 to ≤ 5 and > 5 years), study design (cohort vs. cross‐sectional), geographic region (Asia‐Pacific, Europe, Middle East, North America and others), decade of study (1990s, 2000s, 2010s and 2020s), and *risk of bias* (low vs. moderate/high). Meta‐analyses were also performed separately for gastric and duodenal ulcers. Meta‐regression explored associations of study year (midpoint of the study period), study duration (years), mean age (years), proportion of female participants (%), current smoking (%), alcohol use (%) and previous ulcer history (%) as study‐level moderators. Study year was defined as the midpoint of the study period. When the study period was not reported, the publication year was used as a proxy for study year and decade of study, whereas study period duration was treated as unavailable. Sensitivity analyses included fixed‐effect meta‐analysis, exclusion of unpublished studies, and exclusion of patients with unknown aetiology from the denominator, and analyses restricted to studies with more rigorous or clearly defined methodological characteristics: studies using two or more diagnostic methods for *H. pylori* , studies with a clearly defined NSAID exposure window, studies explicitly classifying aspirin as an NSAID, and studies with a clearly defined study period.

As an ecological analysis, we assigned each study a period‐specific *H. pylori* prevalence estimate based on a previous global meta‐analysis (details in Appendices S1–S12) [16]. Estimates were matched to the study midpoint year and used as an external ecological covariate in meta‐regression. Two exploratory multivariable meta‐regression models were fitted: Model 1 included study year and country‐level *H. pylori* prevalence, and Model 2 was further adjusted for study region.

## 3 Results

### 3.1 Study Selection and Characteristics

**FIGURE 1:**
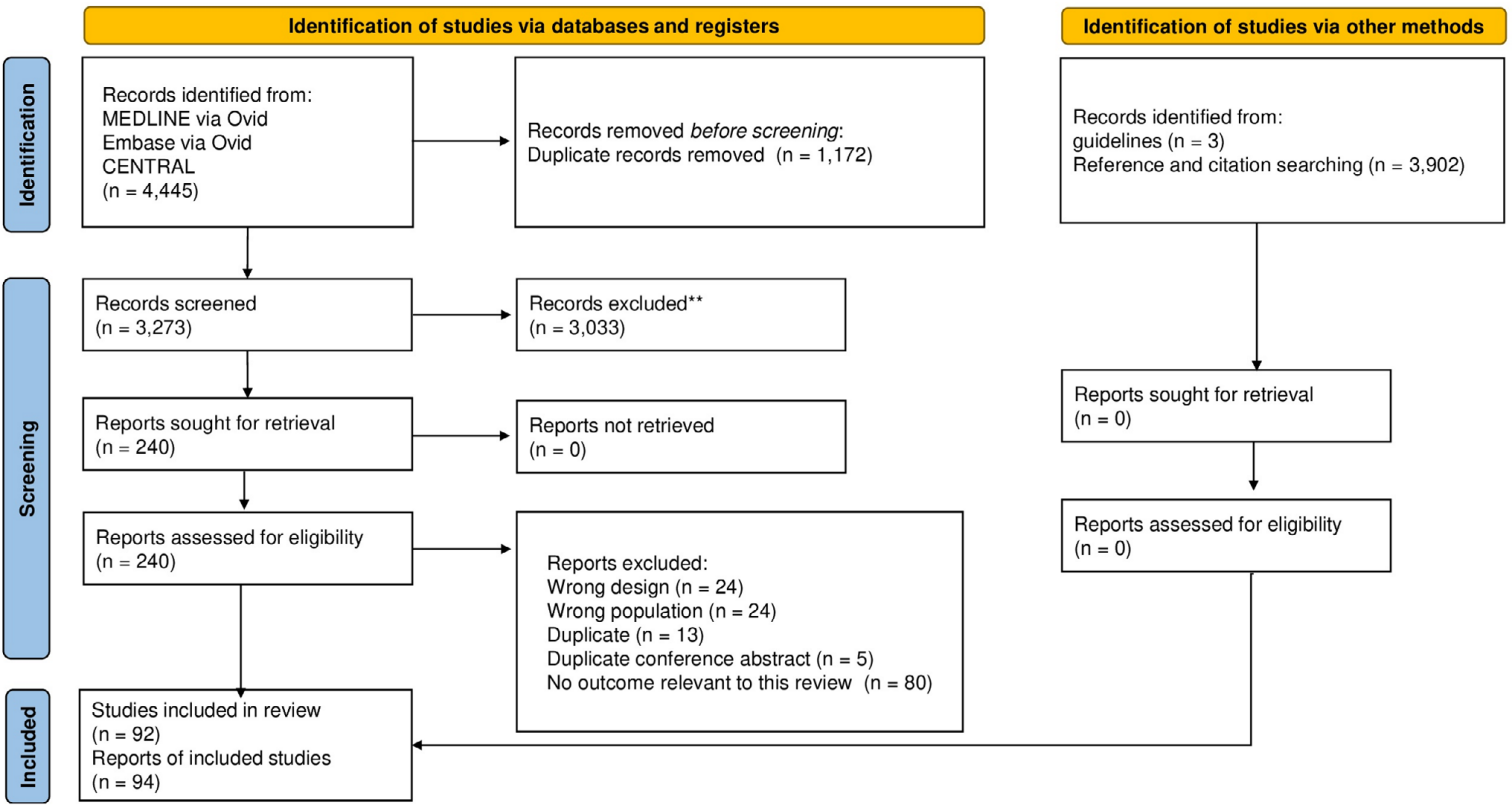
Flow diagram of study selection.

We identified 3273 records (Figure 1) and included 92 studies comprising 60,816 patients from 32 countries (Appendix S5) [17, 18, 19, 20, 21, 22, 23, 24, 25, 26, 27, 28, 29, 30, 31, 32, 33, 34, 35, 36, 37, 38, 39, 40, 41, 42, 43, 44, 45, 46, 47, 48, 49, 50, 51, 52, 53, 54, 55, 56, 57, 58, 59, 60, 61, 62, 63, 64, 65, 66, 67, 68, 69, 70, 71, 72, 73, 74, 75, 76, 77, 78, 79, 80, 81, 82, 83, 84, 85, 86, 87, 88, 89, 90, 91, 92, 93, 94, 95, 96, 97, 98, 99, 100, 101, 102, 103, 104, 105, 106, 107, 108]. Of these, 69 were cross‐sectional and 23 cohort studies. Regarding clinical presentation, 54 studies reported overall PUD, 35 reported bleeding PUD and 5 reported perforated PUD. Overall, 9126 patients were classified as having non‐ *H. pylori* , non‐NSAID PUD. Methods for diagnosing *H. pylori* infection varied and commonly included biopsy, rapid urease test, urea breath test, serology and stool antigen testing, alone or in combination. Definitions of NSAID exposure also differed across studies; when reported, exposure windows ranged from 7 to 90 days before ulcer diagnosis or presentation, and aspirin was often considered an NSAID. The number of studies by study decade was 16 in the 1990s, 25 in the 2000s, 12 in the 2010s and 1 in the 2020s for overall PUD, and 6, 22, 7 and 0, respectively, for bleeding PUD.

Using our prespecified modified overall risk‐of‐bias classification based on the Joanna Briggs Institute Critical Appraisal Tool for prevalence studies, 4 studies were rated low risk, 62 moderate risk and 27 high risk (Appendix S6). Common item‐level concerns included sample frame appropriateness, adequacy of sample coverage, validity of condition identification and response rate, while statistical analyses were considered appropriate in all studies.

### 3.2 Overall PUD

**FIGURE 2:**
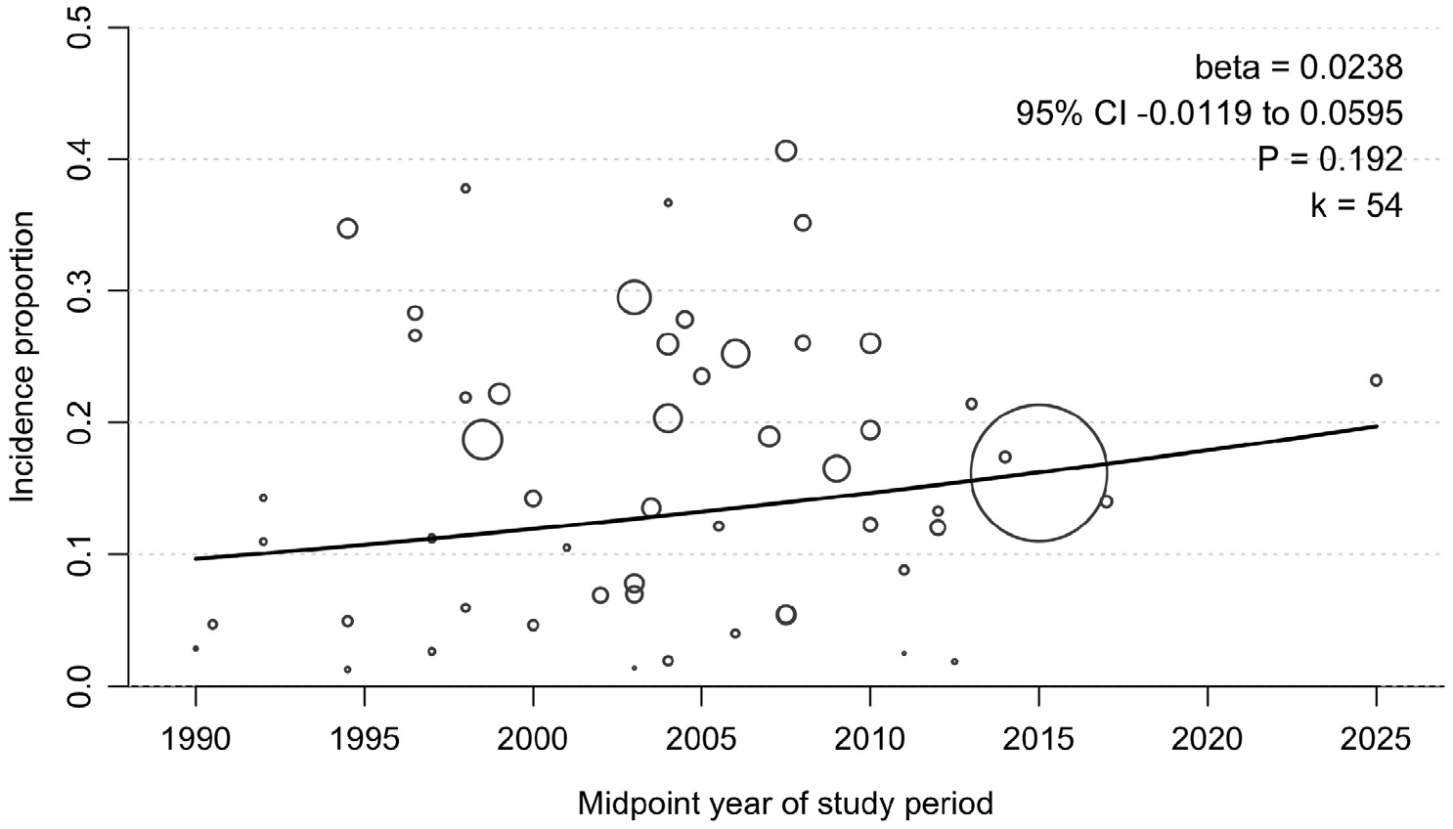
Bubble plot showing the proportion of non‐ *Helicobacter pylori* , non‐NSAID peptic ulcer disease among adults with overall peptic ulcer disease.

Among overall PUD cases, the pooled proportion of non‐ *H. pylori* , non‐NSAID PUD was 13.0% (54 studies; 95% CI, 10.4%–16.2%; 95% PI, 2.4%–47.9%; *I* ^2^ = 98.4%; Table 1). In univariable meta‐regression, study year was not statistically significantly associated with the proportion of non‐ *H. pylori* , non‐NSAID PUD (54 studies; *β* = 0.024 per year; 95% CI, −0.012 to 0.060; *p* = 0.19; Figure 2). Higher proportions of current smokers (18 studies; *β* = 0.052; 95% CI, 0.017–0.086; *p* = 0.003) and alcohol users (12 studies; *β* = 0.023; 95% CI, 0.0002–0.046; *p* = 0.048) were associated with a higher proportion of non‐ *H. pylori* , non‐NSAID PUD (Table 2). In multivariable meta‐regression, study year and country‐level *H. pylori* prevalence were not statistically significantly associated with the proportion of non‐ *H. pylori* , non‐NSAID PUD, and both associations were further attenuated after adjusting for region (Table 3). Subgroup analyses revealed no statistically significant differences by study period duration, study design, geographic region, decade of study, or risk of bias (all *p* > 0.10) (Table 4). Sensitivity analyses produced similar results (Table 5). Site‐specific analyses of gastric and duodenal ulcers are presented in Appendices S7 and S8.

**TABLE 1:** Pooled proportions of etiologic categories among overall and bleeding peptic ulcer disease cases.

Clinical presentation	Etiologic category	*k*	Estimate, %	95% CI^a^, %	95% PI, %
Overall peptic ulcer disease	Non‐ *H. pylori* , non‐NSAID peptic ulcer disease	54	13.0	10.4–16.2	2.4–47.9
	*H. pylori* ‐associated ulcer	42	42.5	35.1–50.2	9.0–84.6
	NSAID‐associated ulcer	41	11.9	9.2–15.3	2.2–44.7
	*H. pylori* and NSAID‐associated ulcer	38	18.0	14.6–21.9	4.7–49.1
Bleeding peptic ulcer disease	Non‐ *H. pylori* , non‐NSAID peptic ulcer disease	35	11.3	8.8–14.4	2.6–38.0
	*H. pylori* ‐associated ulcer	25	29.3	23.4–36.1	8.4–65.3
	NSAID‐associated ulcer	25	22.0	17.9–26.8	7.5–49.7
	*H. pylori* and NSAID‐associated ulcer	24	26.5	20.9–32.9	7.4–61.8

Abbreviations: CI, confidence interval; PI, prediction interval.

^a^ Pooled proportions were estimated using random‐effects models on the logit scale, with between‐study variance estimated by restricted maximum likelihood.

**TABLE 2:** Univariable meta‐regression analyses of study‐level factors associated with the proportion of non‐ *H. pylori* , non‐NSAID peptic ulcer disease in overall and bleeding peptic ulcer disease cases.

Clinical presentation	Moderator^a^	*k*	*p*	Estimates (95% CI)^b^
Overall peptic ulcer disease	Study year, year	54	0.19	0.024 (−0.012 to 0.060)
	Study duration, years	45	0.90	−0.004 (−0.056 to 0.049)
	Age, years	38	0.41	−0.020 (−0.069 to 0.028)
	Female, %	42	0.30	0.014 (−0.012 to 0.040)
	Current smoking, %	18	0.003	0.052 (0.017 to 0.086)
	Alcohol use, %	12	0.048	0.023 (0.0002 to 0.046)
	Previous ulcer disease, %	5	0.13	−0.037 (−0.084 to 0.011)
Bleeding peptic ulcer disease	Study year, year	35	0.0008	0.076 (0.031 to 0.120)
	Study duration, years	33	0.03	−0.105 (−0.201 to −0.008)
	Age, years	26	0.74	0.009 (−0.043 to 0.061)
	Female, %	26	0.72	−0.007 (−0.046 to 0.032)
	Current smoking, %	11	0.60	0.015 (−0.041 to 0.071)
	Alcohol use, %	11	0.39	0.016 (−0.021 to 0.052)
	Previous ulcer disease, %	10	0.17	−0.044 (−0.108 to 0.020)

Abbreviation: CI, confidence interval.

^a^ Study year is defined as the midpoint of the reported study period. Study period duration is the length of the study period in years.

^b^ Estimates represent regression coefficients from univariable random‐effects meta‐regression models on the logit scale.

**TABLE 3:** Multivariable meta‐regression of study‐level factors associated with the proportion of non‐ *H. pylori* , non‐NSAID peptic ulcer disease.

Clinical presentation	Model	*k*	Study year^a^, year *p*	Estimate (95% CI)^a^	*H. pylori* prevalence, % *p*	Estimate (95% CI)^b^
Overall peptic ulcer disease	Model 1	54	0.12	0.028 (−0.008 to 0.063)	0.31	0.020 (−0.018 to 0.058)
	Model 2	54	0.07	−0.016 (−0.033 to 0.001)	0.10	−0.015 (−0.033 to 0.003)
Bleeding peptic ulcer disease	Model 1	35	0.002	0.070 (0.026 to 0.113)	0.07	−0.017 (−0.036 to 0.002)
	Model 2	35	0.009	0.059 (0.015 to 0.103)	0.02	−0.025 (−0.046 to −0.005)

*Note:* Model 1 included study year and country‐level *H. pylori* prevalence. Model 2 additionally adjusted for region.

Abbreviation: CI, confidence interval.

^a^ Study year is defined as the midpoint of the reported study period.

^b^ Estimates are regression coefficients from multivariable random‐effects meta‐regression models on the logit scale.

**TABLE 4:** Subgroup analyses of non‐ *H. pylori* , non‐NSAID peptic ulcer disease proportion among all peptic ulcer disease.

Variable	Subgroup	No. of studies	Pooled proportion, %	95% CI, %	95% PI, %	*p* ^a^
Study period duration	≤ 1 year	14	17.4	12.3–23.9	4.4–48.7	0.51
	1–3 years	16	14.2	9.0–21.6	2.1–56.3	
	3–5 years	7	10.4	4.9–20.6	1.2–52.5	
	> 5 years	8	11.7	6.9–19.1	2.4–41.8	
Study design	Cohort	11	17.4	13.2–22.6	6.5–39.0	0.18
	Cross‐sectional	43	11.9	9.0–15.6	1.8–49.3	
Geographic region	Asia‐Pacific	27	15.9	11.9–20.9	3.5–51.0	0.19
	Europe	16	9.2	5.8–14.2	1.4–41.3	
	Middle East	4	8.4	2.8–22.9	0.7–53.9	
	North America	5	17.8	8.8–32.9	3.0–60.4	
	Other regions	2	11.5	7.6–17.1	6.1–20.7	
Decade of study	1990s	16	11.7	7.0–18.7	1.4–54.9	0.85
	2000s	25	13.6	9.7–18.7	2.3–51.2	
	2010s	12	13.4	9.8–18.1	4.6–33.3	
	2020s	1	23.2	16.3–31.9	—	
Risk of bias	Low	3	9.2	4.3–18.7	2.4–49.1	0.45
	Moderate/High	51	13.3	10.5–16.6	2.1–33.0	

Abbreviations: CI, confidence interval; PI, prediction interval.

^a^ *p* values are for between‐subgroup differences.

**TABLE 5:** Sensitivity analyses of non‐ *H. pylori* , non‐NSAID peptic ulcer disease proportion among all peptic ulcer disease.

Variable	No. of studies	Pooled proportion, %	95% CI, %	95% PI, %	*I* ^2^, %
Main analysis (random‐effect)	54	13.0	10.4–16.2	2.4–47.9	98.4
Main analysis (fixed‐effect)	54	16.9	16.5–17.2	—	95.7
Published studies only (random‐effect)	49	12.2	9.5–15.4	2.1–46.9	98.4
Known aetiology only (random‐effect)	54	13.9	11.1–17.3	2.5–50.5	98.4
Studies using ≥ 2 diagnostic methods for *H. pylori*	37	12.2	9.1–16.1	1.9–49.2	97.7
Studies with a clearly defined NSAID exposure window	27	11.6	8.5–15.8	2.2–44.0	97.6
Studies explicitly classifying aspirin as an NSAID	29	14.5	10.7–19.3	2.6–51.7	97.9
Studies with a clearly defined study period	47	13.0	10.1–16.6	2.2–49.5	98.5

Abbreviations: CI, confidence interval; PI, prediction interval.

### 3.3 Bleeding PUD

**FIGURE 3:**
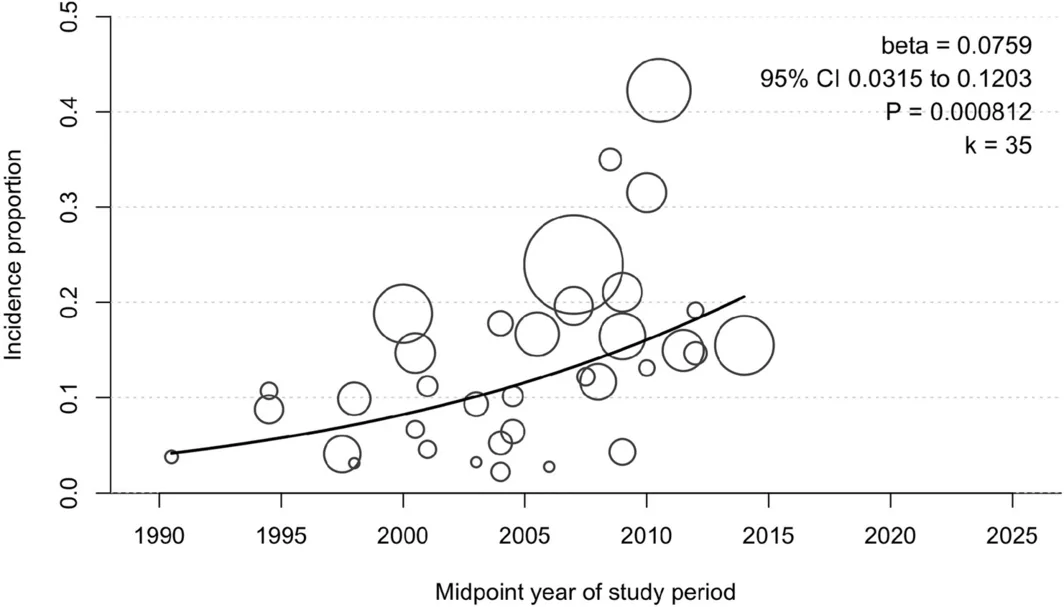
Bubble plot showing the proportion of non‐ *Helicobacter pylori* , non‐NSAID peptic ulcer disease among adults with bleeding peptic ulcer disease.

Among bleeding PUD cases, the pooled proportion of non‐ *H. pylori* , non‐NSAID PUD was 11.3% (35 studies; 95% CI, 8.8%–14.4%; 95% PI, 2.6%–38.0%; *I* ^2^ = 95.5%). Subgroup analyses showed no statistically significant differences by study duration, study design, geographic region, or risk of bias, although proportions varied by decade of study (*p* = 0.004), with higher estimates observed in more recent decades (Table 6). In univariable meta‐regression, a later study year was associated with a higher proportion of non‐ *H. pylori* , non‐NSAID PUD (35 studies; *β* = 0.076; 95% CI, 0.031–0.120; *p* = 0.0008; Figure 3), while longer study duration was associated with a lower proportion (33 studies; *β* = −0.105; 95% CI, −0.201 to −0.008; *p* = 0.03). Sensitivity analyses of the study‐year variable in univariable meta‐regression showed consistent findings (Appendix S9). In univariable meta‐regression restricted to bleeding studies, age, female proportion, smoking, alcohol use and previous ulcer history were not statistically significantly associated with the pooled proportion. In multivariable meta‐regression for bleeding PUD, a later study year remained associated with a higher proportion of non‐ *H. pylori* , non‐NSAID PUD (35 studies; *β* = 0.070; 95% CI, 0.026–0.113; *p* = 0.002). In the model additionally adjusted for region, a later study year (*β* = 0.059; 95% CI, 0.015–0.103; *p* = 0.009) and lower *H. pylori* prevalence (*β* = −0.025; 95% CI, −0.046 to −0.005; *p* = 0.02) were each independently associated with a higher proportion of non‐ *H. pylori* , non‐NSAID bleeding PUD. Sensitivity analyses were broadly consistent (Appendix S10), and site‐specific analyses of bleeding gastric and duodenal ulcers are presented in Appendices S11 and S12.

**TABLE 6:** Subgroup analyses of non‐ *H. pylori* , non‐NSAID peptic ulcer disease proportion among bleeding peptic ulcer disease.

Variable	Subgroup	No. of studies	Pooled proportion, %	95% CI, %	95% PI, %	*p* ^a^
Study period duration	≤ 1 year	9	13.3	8.2–20.7	3.0–42.9	0.46
	1–3 years	12	12.9	8.1–19.8	2.5–46.1	
	3–5 years	6	11.6	7.3–17.8	3.6–31.6	
	> 5 years	6	7.8	4.5–13.1	1.9–26.5	
Study design	Cohort	2	16.3	12.1–21.6	12.1–21.6	0.47
	Cross‐sectional	33	11.0	8.4–14.2	2.4–38.4	
Geographic region	Asia‐Pacific	16	13.6	9.6–18.9	3.2–42.8	0.38
	Europe	13	10.3	6.6–15.9	1.9–40.0	
	Middle East	3	6.1	2.6–13.8	1.3–24.1	
	North America	3	9.4	4.1–20.1	1.9–35.4	
Decade of study	1990s	6	6.5	4.3–9.8	2.5–15.9	0.004
	2000s	22	10.7	7.8–14.3	2.5–35.8	
	2010s	7	20.7	14.0–29.5	7.0–47.8	
Risk of bias	Low	2	7.0	1.3–30.4	0.4–58.3	0.47
	Moderate/High	33	11.5	8.9–14.7	2.6–38.5	

Abbreviations: CI, confidence interval; PI, prediction interval.

^a^ *p* values are for between‐subgroup differences.

### 3.4 Perforated PUD

Among studies on perforated PUD, the pooled proportion of non‐ *H. pylori* , non‐NSAID PUD was 22.0% (5 studies; 95% CI, 13.1%–34.7%; 95% PI, 6.2%–54.5%; *I* ^2^ = 87.1%).

## 4 Discussion

In this systematic review and meta‐analysis of 92 studies including 60,816 patients from 32 countries, we identified 9126 cases of non‐ *H. pylori* , non‐NSAID PUD. The pooled proportions of non‐ *H. pylori* , non‐NSAID PUD were 13.0% for overall PUD, 11.3% for bleeding PUD, and 22.0% for perforated PUD, with wide prediction intervals reflecting substantial between‐study variability. Meta‐regression showed that among overall PUD, higher proportions of current smoking and alcohol use may be associated with a greater prevalence of non‐ *H. pylori* , non‐NSAID PUD, whereas in bleeding PUD, a more recent study year may be associated with a higher proportion. In exploratory ecological multivariable meta‐regression using country‐level estimates of *H. pylori* prevalence, the temporal association in overall PUD was partly attenuated after accounting for country‐level *H. pylori* prevalence, whereas in bleeding PUD, the association with a more recent study year persisted after adjustment, and lower *H. pylori* prevalence also remained associated with a higher proportion after adjusting for region. These findings suggest that non‐ *H. pylori* , non‐NSAID PUD represents a non‐negligible but heterogeneous component of PUD. Temporal patterns in bleeding PUD may reflect evolving etiologic patterns beyond *H. pylori* infection and NSAID exposure.

The observed association with study‐level proportions of current smoking and alcohol use raises the possibility that lifestyle‐related factors may be associated with non‐ *H. pylori* , non‐NSAID PUD. In the absence of *H. pylori* infection or NSAID exposure, ulcers may develop through alternative mechanisms, including impaired mucosal defence, microvascular compromise, oxidative stress and delayed ulcer healing [109]. However, these results should be interpreted as exploratory because the meta‐regression analyses were based on aggregate study‐level data, included a limited number of studies for some covariates, and were not adjusted for multiple testing. Therefore, these analyses cannot establish individual‐level associations or causal relationships [110]. These findings support the possibility that non‐ *H. pylori* , non‐NSAID PUD may represent, at least in part, a distinct clinical phenotype with risk factors beyond the conventional framework of *H. pylori* infection and NSAID exposure.

Among bleeding PUD, the increasing proportion of non‐ *H. pylori* , non‐NSAID PUD over time should be interpreted cautiously, as our analysis was based on proportions rather than incidence. We have limited data in more recent years and so do not have sufficient information on time trends in the 2020s. The temporal association may also be influenced by temporal misclassification, changes in *H. pylori* testing, differences in ascertainment of NSAID and aspirin exposure, and residual ecological confounding. Nevertheless, the direction of the association was not materially changed in methodologically restricted sensitivity analyses addressing study‐period definition, *H. pylori* diagnostic assessment, and NSAID/aspirin exposure definitions. In addition, the association with more recent study year persisted after adjustment for background *H. pylori* prevalence. These findings suggest that declining *H. pylori* prevalence alone does not fully explain the temporal trend. Contemporary prospective studies using standardized definitions of *H. pylori* infection, NSAID and aspirin exposure, study period, and unknown aetiology are needed to define the current burden of non‐ *H. pylori* , non‐NSAID PUD.

Although not fully accounted for in our analyses, previous studies indicate that substantial systemic comorbidity may contribute. Non‐ *H. pylori* , non‐NSAID PUD often occurs in patients with substantial comorbidities, including end‐stage renal disease, liver cirrhosis and advanced vascular disease [10, 11, 111]. These characteristics overlap with host profiles recognized in contemporary high‐bleeding risk frameworks, particularly advanced age, renal dysfunction and vascular disease [112, 113]. In this context, our findings suggest that the residual temporal trend partly reflects factors related to substantial systemic comorbidity beyond the traditional framework of *H. pylori* infection and NSAID use alone. Previous studies have indicated that non‐ *H. pylori* , non‐NSAID PUD may be associated with lower healing rates and higher risks of recurrence or rebleeding than other etiologic subtypes [11, 46, 106]. Therefore, a broader clinical perspective may be warranted, considering non‐ *H. pylori* , non‐NSAID PUD in the context of systemic comorbidity and potentially competing bleeding and thrombotic/ischemic risks.

This study has several limitations. First, there was substantial clinical and methodological variability across studies, including differences in ulcer presentation, definitions of NSAID exposure, and ascertainment of *H. pylori* infection. Prespecified subgroup analyses, meta‐regression, and post hoc sensitivity analyses, however, supported the overall robustness of our findings. Second, some non‐ *H. pylori* , non‐NSAID ulcers may have been misclassified because *H. pylori* testing can yield false negatives during acute bleeding or proton pump inhibitor use, and non‐NSAID ulcerogenic medications were not consistently captured. Nevertheless, these factors are unlikely to fully account for the observed burden of non‐ *H. pylori* , non‐NSAID PUD. Third, estimates for other etiologic categories were available only in a subset of studies and should be considered exploratory. Finally, the limited number of national cohort studies may restrict generalizability. Large, prospective, geographically diverse studies are needed to clarify the epidemiology, risk factors, and clinical significance of non‐ *H. pylori* , non‐NSAID PUD.

## 5 Conclusions

This systematic review and meta‐analysis demonstrates that non‐ *H. pylori* , non‐NSAID PUD is a non‐negligible but heterogeneous component of contemporary PUD. In bleeding PUD, exploratory ecological meta‐regression suggested the higher proportion observed in more recent studies persisted after adjustment for country‐level *H. pylori* prevalence, and lower *H. pylori* prevalence remained associated with a higher proportion after additional adjustment for region. These findings may reflect evolving an etiologic profile of PUD beyond the traditional framework of *H. pylori* infection and NSAID exposure. Large, prospective studies with standardized ascertainment of *H. pylori* status and medication exposure are needed to better define its epidemiology and risk factors.

## Author Contributions

**Tomoari Kamada:** writing – review and editing, supervision. **Hiromi Sekiguchi:** conceptualization, investigation, writing – original draft, resources, data curation, validation, formal analysis. **Paul Moayyedi:** methodology, validation, visualization, writing – review and editing. **Jun Watanabe:** conceptualization, methodology, software, data curation, resources, investigation, writing – original draft, visualization, validation, formal analysis, supervision. **Satoshi Sato:** data curation, writing – review and editing, validation, investigation. **Keigo Misawa:** data curation, writing – review and editing, validation, investigation. **Atsushi Masamune:** writing – review and editing, supervision. **Tomonori Yano:** writing – review and editing, supervision. **Takeshi Kanno:** project administration, conceptualization, writing – review and editing, investigation, validation, supervision, data curation, formal analysis, resources. **Yuhong Yuan:** methodology, writing – review and editing, visualization, validation, formal analysis, software.

## Funding

The authors have nothing to report.

## Conflicts of Interest

Jun Watanabe received financial support for overseas study, unrelated to this research, from the following organizations: The Uehara Memorial Foundation, KOSÉ Cosmetology Research Foundation, Grant of The Clinical Research Promotion Foundation 2025. All other authors declare no conflicts of interest.

## Supporting information


**Appendix S1:** PRISMA checklist.
**Appendix S2:** Amendments from the registered protocol.
**Appendix S3:** Detailed methods.
**Appendix S4:** Search strategy.
**Appendix S5:** Overview of baseline characteristics.
**Appendix S6:** Joanna Briggs Institute Critical Appraisal Checklist for Studies Reporting Prevalence Data.
**Appendix S7:** Subgroup analyses of non‐ *H. pylori* , non‐NSAID peptic ulcer disease proportion among gastric peptic ulcer disease.
**Appendix S8:** Subgroup analyses of non‐ *H. pylori* , non‐NSAID peptic ulcer disease proportion among duodenal peptic ulcer disease.
**Appendix S9:** Sensitivity analyses of study in univariable meta‐regression analyses of study‐level factors associated with the proportion of non‐ *H. pylori* , non‐NSAID peptic ulcer disease in bleeding peptic ulcer disease cases.
**Appendix S10:** Sensitivity analyses of non‐ *H. pylori* , non‐NSAID peptic ulcer disease proportion among bleeding peptic ulcer disease.
**Appendix S11:** Subgroup analyses of non‐ *H. pylori* , non‐NSAID peptic ulcer disease proportion among bleeding gastric peptic ulcer disease.
**Appendix S12:** Subgroup analyses of non‐ *H. pylori* , non‐NSAID peptic ulcer disease proportion among bleeding duodenal peptic ulcer disease.

## Data Availability

The extracted study‐level dataset and R code used for the meta‐analyses are publicly on the Open Science Framework at https://doi.org/10.17605/OSF.IO/5B27R.
