# Supplementary material for: Meta‐Analysis: Prevalence of Non‐ Helicobacter pylori , Non‐NSAID Peptic Ulcer Disease
Source: Aliment Pharmacol Ther. 2026 Aug 10;64(8):1034–45. doi: 10.1111/apt.70906 (PMC13603966; doi:10.1111/apt.70906)
Supplement: Supplementary file 1 [file APT-64-1034-s001.docx]

**Online Supplementary materials**

**Appendix S1:** PRISMA checklist

**Appendix S2:** Amendments from the registered protocol

**Appendix S3:** Detailed Methods

**Appendix S4:** Search strategy

**Appendix S5.** Overview of baseline characteristics

**Appendix S6:** Joanna Briggs Institute Critical Appraisal Checklist for Studies Reporting Prevalence Data

**Appendix S7.** Subgroup analyses of non-*H. pylori*, non-NSAID peptic ulcer disease proportion among gastric peptic ulcer disease

**Appendix S8.** Subgroup analyses of non-*H. pylori*, non-NSAID peptic ulcer disease proportion among duodenal peptic ulcer disease

**Appendix S9.** Sensitivity analyses of study in univariable meta-regression analyses of study-level factors associated with the proportion of non-*H. pylori*, non-NSAID peptic ulcer disease in bleeding peptic ulcer disease cases

**Appendix S10.** Sensitivity analyses of non-H. pylori, non-NSAID peptic ulcer disease proportion among bleeding peptic ulcer disease

**Appendix S11.** Subgroup analyses of non-*H. pylori*, non-NSAID peptic ulcer disease proportion among bleeding gastric peptic ulcer disease

**Appendix S12.** Subgroup analyses of non-*H. pylori*, non-NSAID peptic ulcer disease proportion among bleeding duodenal peptic ulcer disease

**Appendix S1:** PRISMA checklist

| **Section and Topic** | **Item #** | **Checklist item** | **Location where item is reported** |
| --- | --- | --- | --- |
| **TITLE** | | |  |
| Title | 1 | Identify the report as a systematic review. | 1 |
| **ABSTRACT** | | |  |
| Abstract | 2 | See the PRISMA 2020 for Abstracts checklist. | 3 |
| **INTRODUCTION** | | |  |
| Rationale | 3 | Describe the rationale for the review in the context of existing knowledge. | 5 |
| Objectives | 4 | Provide an explicit statement of the objective(s) or question(s) the review addresses. | 5 |
| **METHODS** | | |  |
| Eligibility criteria | 5 | Specify the inclusion and exclusion criteria for the review and how studies were grouped for the syntheses. | 6 |
| Information sources | 6 | Specify all databases, registers, websites, organisations, reference lists and other sources searched or consulted to identify studies. Specify the date when each source was last searched or consulted. | 7 |
| Search strategy | 7 | Present the full search strategies for all databases, registers and websites, including any filters and limits used. | Supplement material C |
| Selection process | 8 | Specify the methods used to decide whether a study met the inclusion criteria of the review, including how many reviewers screened each record and each report retrieved, whether they worked independently, and if applicable, details of automation tools used in the process. | 7 |
| Data collection process | 9 | Specify the methods used to collect data from reports, including how many reviewers collected data from each report, whether they worked independently, any processes for obtaining or confirming data from study investigators, and if applicable, details of automation tools used in the process. | 7 |
| Data items | 10a | List and define all outcomes for which data were sought. Specify whether all results that were compatible with each outcome domain in each study were sought (e.g. for all measures, time points, analyses), and if not, the methods used to decide which results to collect. | 7 |
|  | 10b | List and define all other variables for which data were sought (e.g. participant and intervention characteristics, funding sources). Describe any assumptions made about any missing or unclear information. | 7 |
| Study risk of bias assessment | 11 | Specify the methods used to assess risk of bias in the included studies, including details of the tool(s) used, how many reviewers assessed each study and whether they worked independently, and if applicable, details of automation tools used in the process. | 7 |
| Effect measures | 12 | Specify for each outcome the effect measure(s) (e.g. risk ratio, mean difference) used in the synthesis or presentation of results. | 7, 8 |
| Synthesis methods | 13a | Describe the processes used to decide which studies were eligible for each synthesis (e.g. tabulating the study intervention characteristics and comparing against the planned groups for each synthesis (item #5)). | 7-9 |
|  | 13b | Describe any methods required to prepare the data for presentation or synthesis, such as handling of missing summary statistics, or data conversions. | 7-9 |
|  | 13c | Describe any methods used to tabulate or visually display results of individual studies and syntheses. | 7-9 |
|  | 13d | Describe any methods used to synthesize results and provide a rationale for the choice(s). If meta-analysis was performed, describe the model(s), method(s) to identify the presence and extent of statistical heterogeneity, and software package(s) used. | 7-9 |
|  | 13e | Describe any methods used to explore possible causes of heterogeneity among study results (e.g. subgroup analysis, meta-regression). | 7-9 |
|  | 13f | Describe any sensitivity analyses conducted to assess robustness of the synthesized results. | 7-9 |
| Reporting bias assessment | 14 | Describe any methods used to assess risk of bias due to missing results in a synthesis (arising from reporting biases). | - |
| Certainty assessment | 15 | Describe any methods used to assess certainty (or confidence) in the body of evidence for an outcome. | - |
| **RESULTS** | | |  |
| Study selection | 16a | Describe the results of the search and selection process, from the number of records identified in the search to the number of studies included in the review, ideally using a flow diagram. | 10 |
|  | 16b | Cite studies that might appear to meet the inclusion criteria, but which were excluded, and explain why they were excluded. | 10 |
| Study characteristics | 17 | Cite each included study and present its characteristics. | 10, Appendix S5 |
| Risk of bias in studies | 18 | Present assessments of risk of bias for each included study. | 10, 11, Appendix S6 |
| Results of individual studies | 19 | For all outcomes, present, for each study: (a) summary statistics for each group (where appropriate) and (b) an effect estimate and its precision (e.g. confidence/credible interval), ideally using structured tables or plots. | 10, Appendix S5 |
| Results of syntheses | 20a | For each synthesis, briefly summarise the characteristics and risk of bias among contributing studies. | 11–13 |
|  | 20b | Present results of all statistical syntheses conducted. If meta-analysis was done, present for each the summary estimate and its precision (e.g. confidence/credible interval) and measures of statistical heterogeneity. If comparing groups, describe the direction of the effect. | 11–13 |
|  | 20c | Present results of all investigations of possible causes of heterogeneity among study results. | 11–13 |
|  | 20d | Present results of all sensitivity analyses conducted to assess the robustness of the synthesized results. | 11–13 |
| Reporting biases | 21 | Present assessments of risk of bias due to missing results (arising from reporting biases) for each synthesis assessed. | - |
| Certainty of evidence | 22 | Present assessments of certainty (or confidence) in the body of evidence for each outcome assessed. | - |
| **DISCUSSION** | | |  |
| Discussion | 23a | Provide a general interpretation of the results in the context of other evidence. | 13, 14 |
|  | 23b | Discuss any limitations of the evidence included in the review. | 17, 18 |
|  | 23c | Discuss any limitations of the review processes used. | 17, 18 |
|  | 23d | Discuss implications of the results for practice, policy, and future research. | 17, 18 |
| **OTHER INFORMATION** | | |  |
| Registration and protocol | 24a | Provide registration information for the review, including register name and registration number, or state that the review was not registered. | 6 |
|  | 24b | Indicate where the review protocol can be accessed, or state that a protocol was not prepared. | 6 |
|  | 24c | Describe and explain any amendments to information provided at registration or in the protocol. | Appendix S2 |
| Support | 25 | Describe sources of financial or non-financial support for the review, and the role of the funders or sponsors in the review. | 19 |
| Competing interests | 26 | Declare any competing interests of review authors. | 19 |
| Availability of data, code and other materials | 27 | Report which of the following are publicly available and where they can be found: template data collection forms; data extracted from included studies; data used for all analyses; analytic code; any other materials used in the review. | 19 |

**Appendix S2:** Amendments from the registered protocol

1. Heterogeneity

In the protocol, heterogeneity was described using prespecified I. thresholds. In the final review, these thresholds were not used as formal decision rules because high I. values are common in meta-analyses of proportions. Heterogeneity was therefore interpreted in the context of the spread of study-specific estimates and, where applicable, subgroup analyses and meta-regression.

2. The modified Joanna Briggs Institute Prevalence Critical Appraisal Tool

Two reviewers will independently assess risk of bias using the Joanna Briggs Institute Prevalence Critical Appraisal Tool. Each item will be judged as Yes, No, or Unclear. As the JBI tool does not generally recommend deriving a single overall risk-of-bias judgment, we adapted the tool for the purposes of this review and applied prespecified decision rules to assign an overall study-level rating. Studies with all items rated Yes will be considered low risk of bias, those with up to four No and/or Unclear ratings will be considered moderate risk of bias, and those with five or more No and/or Unclear ratings will be considered high risk of bias. Disagreements will be resolved by discussion, with a third reviewer acting as arbiter if required.

3. Meta-analysis

Although the protocol prespecified a DerSimonian–Laird random-effects model with Freeman–Tukey double-arcsine transformation, the final analysis used logittransformed proportions with restricted maximum-likelihood estimation in R (metafor).

This modification was made to provide a consistent framework for the primary metaanalysis, meta-regression, and sensitivity analyses, and to avoid known interpretational issues related to back-transformation of Freeman–Tukey estimates.

4. Subgroup analyses and meta-regression

Compared with the original protocol, the planned subgroup analysis according to ulcer type was replaced by separate restricted meta-analyses for gastric ulcer only and duodenal ulcer only. We additionally performed post hoc subgroup analyses according to study period duration (≤1 year, 1–3 years, 3–5 years, and >5 years) and exploratory univariable meta-regression analyses using study period duration (years), age (years), female proportion (%), current smoking (%), alcohol use (%), and previous ulcer disease (%) as study-level moderators. These analyses were added to further explore potential sources of between-study heterogeneity.

5. Sensitivity analysis

The main analysis included conference abstracts when eligible, and we performed a sensitivity analysis restricted to published studies only. We additionally performed a sensitivity analysis excluding patients with unknown etiology from the denominator. We also analyzed restricted to studies with more rigorous or clearly defined methodological characteristics: studies using two or more diagnostic methods for *H. pylori*, studies with a clearly defined NSAID exposure window, studies explicitly classifying aspirin as an NSAID, and studies with a clearly defined study period.

6. Multivariable meta-regression analyses of study-level factors associated with the proportion of non-H. pylori, non-NSAID peptic ulcer disease

As background H. pylori prevalence was unavailable in most primary studies, we derived external proxy estimates for use in additional ecological meta-regression analyses, using the global meta-analysis by Chen et al (Gastroenterology. 2024;166(4):605-619). Specifically, for each study, we identified the study midpoint year and matched it to the corresponding period in the report by Chen et al. We then visually approximated adult H. pylori prevalence from the published secular trend curves: country-specific estimates were obtained from the country-level curves in Supplementary Figure 3 whenever available, and otherwise from the regional curves in Figure 2 after assigning the study country to the appropriate World Health Organization region. When necessary, countries were reclassified to align with the regional definitions used by Chen et al. For studies conducted beyond the most recent period reported in that meta-analysis, the nearest available period estimate was used. The resulting values were treated as ecological covariates and incorporated into random-effects meta-regression models of the logit-transformed proportion. Model 1 included study midpoint year and background H. pylori prevalence, and Model 2 further adjusted for study region as a categorical moderator. Because these prevalence estimates were visually derived proxy measures from an external source, they were considered approximate ecological indicators and were used in sensitivity analyses rather than as exact study-level prevalence measurements.

**Appendix S3:** Detailed Methods

**METHODS**

**Protocol**

We followed the Preferred reporting items for systematic review and meta-analysis 2020 (PRISMA-2020). We registered this protocol in PROSPERO (CRD42020211347) (Appendices S1 and S2).

**Study selection**

We included observational (prevalence) studies that assessed the proportion of non-*H. pylori*, non-NSAID PUD among adults with overall PUD, bleeding PUD, and perforated PUD. We excluded RCTs, case reports, case series, review articles, and letters. We included adult patients aged ≥ 18 years who were diagnosed with gastric and/or duodenal ulcers. We excluded studies that did not report the proportion of non-*H. pylori*, non-NSAID PUD, or provide sufficient data to calculate it.

**Types of outcome measures**

The primary outcome was the proportion of non-*H. pylori*, non-NSAID PUD among adults with overall PUD, bleeding PUD, and perforated PUD.

**Electronic searches**

We conducted a literature search to identify observational studies. The literature search identified potential studies in all languages. We translated non-English language studies and fully assessed for potential inclusion in the review as necessary. We did not apply language or country restriction. The search strategies are given in the appendices. We conducted the initial literature search on September 26, 2025, followed by an updated search on March 21, 2026. For identifying potential studies, the following electronic search databases were used:

• Cochrane Central Register of Controlled Trials (CENTRAL) (from 1990 to March 21, 2026),

• MEDLINE (from 1990 to March 21, 2026),

• EMBASE (from 1990 to March 21, 2026).

We checked the reference lists of the international guidelines by the Japanese Society of Gastroenterology, the American Gastroenterological Association, and the European Society of Gastrointestinal Endoscopy. We further hand-searched the reference and cited lists of the studies identified in the above search to identify additional relevant studies.

**Data collection**

Two authors (JW and HS) independently screened the titles and abstracts for eligibility. We retrieved the full texts of potentially eligible studies and two authors independently screened the full text, identified studies for inclusion, and identified and recorded reasons for exclusion of ineligible studies. We assessed each study for potential duplicate publication. When the authors were unable to reach a consensus, the senior author (TK) arbitrated. The same two review authors retrieved and reviewed complete reports of all selected articles.

Two review authors (JW and HS) independently extracted the study and patient characteristics. We resolved any disagreement by consensus with a third review author (TK).

Two review authors (JW and HS) independently used the Joanna Briggs Institute Prevalence Critical Appraisal Tool to assess the methodological quality of the included studies. We resolved disagreement by discussion and consensus with a third review author (TK). For subgroup analyses, we applied decision rules to classify studies as having low, moderate, or high risk of bias (details are provided in the Supplementary Material). We contacted the original authors for further clarification as necessary.

**Statistical Analysis**

We assessed statistical heterogeneity using the I2 statistic. Because high I2 values are common in meta-analyses of proportions, we did not interpret heterogeneity using fixed I^2^ thresholds alone. Instead, we explored heterogeneity through subgroup analyses and meta-regression.

We did not examine a funnel plot and Egger’s test because their assumptions and operating characteristics have not been validated for proportional outcomes.

We performed a meta-analysis of logit-transformed proportions using restricted maximum-likelihood estimation. Pooled estimates and 95% confidence intervals (CIs) were back-transformed and presented as percentages. We used the random‐effects model by default. The random‐effects method incorporates an assumption that underlying effects follow a normal distribution. We expressed dichotomous outcomes as estimates of proportion of patients with non-*H. pylori*, non-NSAID PUD among adults with overall PUD, bleeding PUD, and perforated PUD. We additionally estimated the pooled proportions of *H. pylori* ulcer-associated ulcer, NSAID-associated ulcer, and ulcer associated with both *H. pylori* infection and NSAID use among all PUD cases and, separately, among bleeding PUD cases. We contacted authors for any missing data from the included studies.

We evaluated potential sources of heterogeneity as planned a priori by subgroup analysis—study period duration (≤1 year vs >1 to ≤3 years vs >3 to ≤5 years vs >5years), study design (cohort vs cross-sectional), geographic region (Asia-Pacific vs

Europe vs Middle East vs North America vs others), decade of study (1990s vs 2000s vs

2010s vs 2020s), and Risk of bias (low vs moderate/high). We performed meta-analyses

separately for gastric ulcer only and duodenal ulcer only. We performed metaregression

to explore associations with study year (year), defined as the midpoint of the

study period, study period duration (years), defined as the number of years covered by

each study, age (years), female proportion (%), current smoking (%), alcohol use (%),

and previous ulcer disease (%) as study-level moderators.

We planned to perform sensitivity analyses to assess the robustness of our conclusions

by performing fixed-effect meta-analysis, excluding unpublished studies, and excluding

patients with unknown etiology from the denominator.

As ecological analyses, we assigned each study a period-specific background *H. pylori*

prevalence estimate based on a previous global meta-analysis (details are provided in

the Supplementary Material).16 Estimates were matched according to study midpoint

year and entered as an external ecological covariate in meta-regression analyses. We

fitted two exploratory multivariable meta-regression models: Model 1 included study

year and country-level background *H. pylori* prevalence, and Model 2 additionally

adjusted for study region.

**Appendix S4:** Search strategy

The following search strategy was used from inception until March 21, 2026.

Database: EBM Reviews - Cochrane Central Register of Controlled Trials <March 2026>, Embase <1974 to March 21, 2026>, Ovid MEDLINE(R) ALL <1946 to March 21, 2026>

1 (idiopathic adj5 (ulcer* or PUD or PUDs)).ti,ab,kw,kf.

2 (("non" or "no" or "not" or "negative") adj10 (NSAID or NSAIDs or ((nonsteroid* or

non steroid*) adj (antiinflammator* or anti inflammator*))) adj10 (Helicobacter or

pylori or Hp or "H.pylori" or "H. pylori") adj10 (ulcer* or PUD or PUDs)).ti,ab,kw,kf.

3 or/1-2 [set1- non-HP non-NSAIDs PUD]

4 exp peptic ulcer/

5 exp peptic ulcer hemorrhage/

6 exp peptic ulcer perforation/

7 exp duodenal ulcer/

8 exp stomach ulcer/

9 ((peptic* or stomach or duoden* or gastric or gastro* or esophag* or oesophag* or

PUD) adj3 ulcer$).ti,ab,kw,kf.

10 or/4-9 [PUD]

11 exp Helicobacter/

12 exp Helicobacter Infections/

13 (helicobacter or campylobacter).ti,ab,kw,kf.

14 (pylori or "H.pylori" or "H. pylori" or pyloridis or HP).ti,ab,kw,kf.

15 or/11-14 [HP]

16 exp Anti-Inflammatory Agents, Non-Steroidal/

17 ((non?steroid* or non steroid*) adj3 (anti?inflammator* or anti

inflammator*)).ti,ab,kw,kf.

18 NSAID*.ti,ab,kw,kf.

19 (aspirin* or acetylsalicylic* acid*).ti,ab,kw,kf. or Aspirin/

20 apazone*.ti,ab,kw,kf. or Apazone/

21 celecoxib*.ti,ab,kw,kf. or Celecoxib/

22 diclofenac*.ti,ab,kw,kf. or Diclofenac/

23 diflunisal*.ti,ab,kw,kf. or Diflunisal/

24 (epirizole or etodolac*).ti,ab,kw,kf. or Etodolac/

25 fenoprofen*.ti,ab,kw,kf. or Fenoprofen/

26 flurbiprofen*.ti,ab,kw,kf. or Flurbiprofen/

27 ibuprofen*.ti,ab,kw,kf. or Ibuprofen/

28 indomethacin*.ti,ab,kw,kf. or Indomethacin/

29 ketoprofen*.ti,ab,kw,kf. or Ketoprofen/

30 Ketorolac/ or Ketorolac Tromethamine/ or ketorolac*.ti,ab,kw,kf.

31 (meclofenamic acid* or mefenamic acid* or mesalamine*).ti,ab,kw,kf. or

Meclofenamic Acid/

32 meclofenamate*.ti,ab,kw,kf.

33 meloxicam*.ti,ab,kw,kf. or Meloxicam/

34 (methyl salicylate or methylsalicylate or morniflumate*).ti,ab,kw,kf.

35 nabumetone*.ti,ab,kw,kf. or Nabumetone/

36 naproxen*.ti,ab,kw,kf. or Naproxen/

37 (niflumic* acid* or nimesulide*).ti,ab,kw,kf. or Niflumic Acid/

38 (oxaprozin* or oxyphenbutazone).ti,ab,kw,kf. or Oxaprozin/

39 phenylbutazone*.ti,ab,kw,kf. or Phenylbutazone/

40 piroxicam*.ti,ab,kw,kf. or Piroxicam/

41 salicylate*.ti,ab,kw,kf. or Salicylates/

42 (sulindac* or suprofen).ti,ab,kw,kf. or Sulindac/

43 tenoxicam*.ti,ab,kw,kf.

44 tolmetin*.ti,ab,kw,kf. or Tolmetin/

45 tiaprofenic acid*.ti,ab,kw,kf.

46 exp Cyclooxygenase Inhibitors/

47 celecoxib.ti,ab,kw,kf.

48 or/16-47 [NSAIDs]

49 10 and 15 and 48 [PUD and HP and NSAIDs]

50 prevalence/

51 incidence/

52 (prevalence* or incidence*).ti,ab,kw,kf.

53 (proportion* or percentage*).ti,ab,kw,kf.

54 exp epidemiology/

55 epidemiolog*.ti,ab,kw,kf.

56 or/50-55 [prevalence]

57 49 and 56 [set 2- PUD and HP and NSAIDs and prevalence]

58 3 or 57 [two sets of non-Hp non-NSAIDs PUD]

59 case report/ or case repors/ or (case report or case reports or case series or a case of

or a rare case).ti.

60 58 not 59

61 (child/ or Pediatrics/ or Adolescent/ or Infant/ or adolescence/ or newborn/) not

(adult/ or aged/)

62 ((baby or babies or child or children or pediatric* or paediatric* or peadiatric* or

infant* or infancy or neonat* or newborn* or new born* or adolescen* or preschool or

pre-school or toddler*) not (aged or adult* or elder* or senior* or men or women)).tw.

63 60 not (61 or 62)

64 limit 63 to yr="1990 -Current"

65 remove duplicates from 64

**Appendix S5.** Overview of baseline characteristics

| Authors, year | Country | Study design | Recruitment period | Clinical denominator | Total patients, n | Female, % | Age (year) | Total number of non-HP, non-NSAID peptic ulcer, n (%) | *H. pylori* diagnostic methods | | | | | NSAID definition | |
| --- | --- | --- | --- | --- | --- | --- | --- | --- | --- | --- | --- | --- | --- | --- | --- |
|  |  |  |  |  |  |  |  |  | Biopsy | Rapid urease test | Urea breath test | Serology (IgG) | Stool antigen test | NSAID exposure window | Aspirin included in NSAIDs |
| Antunes, 2016 | Portugal | cross-sectional | 2009-2014 | Bleeding | 381 | NR | 71.0 | 57 | Unclear | Unclear | Unclear | Unclear | Unclear | NR | NR |
| Aoyama, 2000 | Japan | cross-sectional | 1996-1998 | All | 302 | NR | NR | 8 | Yes | No | No | Yes | No | NR | Exclude |
| Archampong, 2016 | Ghana | cross-sectional | 2010-2012 | All | 170 | 47.5 | 51.4 | 15 | No | Yes | No | No | No | NR | NR |
| Arents, 2004 | Netherlands | cohort | 1991-1998 | All | 405 | NR | NR | 20 | Yes | Yes | No | No | No | NR | NR |
| Arroyo, 2004 | Spain | cross-sectional | NR | All | 830 | 31.0 | 58.0 | 16 | Yes | Yes | Yes | No | No | 30 | Include |
| Auwyang, 2013 | Indonesia | cross-sectional | NR | All | 112 | NR | NR | 24 | Yes | Yes | No | No | No | NR | NR |
| Bobrzynski, 2002 | Poland | cross-sectional | 1996-2001 | Bleeding | 135 | 33.3 | 64.0 | 9 | No | No | Yes | No | No | NR | NR |
| Borody, 1991 | Australia | cross-sectional | NR | Duodenal ulcer | 302 | NR | NR | 10 | Unclear | Unclear | Unclear | Unclear | Unclear |  |  |
| Bytzer, 2001 | Denmark | cross-sectional | 1993-1995 | Duodenal ulcer | 276 | 29.1 | 54.0 | 25 | Yes | No | Yes | No | No | NR | Include |
| Carli, 2015 | Brasil | cohort | 1997-2010 | All | 540 | 37.0 | 54.4 | 73 | Yes | No | No | No | No | NR | Include |
| Cekin, 2012 | Turkey | cross-sectional | 2009-2011 | Duodenal ulcer | 152 | NR | 41.0 | 9 | Yes | Yes | No | No | No | NR | Include |
| Chan, 2001 | Hong Kong | cohort | 1997-1998 | Bleeding | 977 | 31.3 | 69.0 | 40 | Yes | Yes | No | No | No | 28 | Include |
| Charpignon, 2013 | France | cross-sectional | 2009-2010 | All | 933 | 42.1 | 61.8 | 154 | Yes | No | Yes | Yes | No | 30 | Include |
| Chason, 2013 | USA | cross-sectional | 2006-2012 | Bleeding | 444 | 31.8 | 53.7 | 63 | Yes | Yes | No | Yes | Yes | 28 | Include |
| Chautems, 1998 | Switzerland | cross-sectional | 1994-1995 | Bleeding | 84 | 39.3 | 69.0 | 9 | Yes | Yes | No | No | No | recent | Include |
| Chen, 2010 | Taiwan | cross-sectional | 2003-2004 | All | 731 | 29.0 | 56.1 | 51 | Yes | No | Yes | No | No | 28 | Include |
| Chiorean, 2002 | USA | cross-sectional | 1984-1997 | All | 298 | NR | NR | 14 | Unclear | Unclear | Unclear | Unclear | Unclear | 14 | Include |
| Chng,2011 | Singapore | cross-sectional | 2007-2008 | Bleeding | 47 | 19.2 | 62.0 | 9 | Yes | Yes | No | Yes | No | 90 | NR |
| Choi, 2023 | South Korea | cross-sectional | 2010-2020 | All | 26785 | 39.9 | NR | 4333 | No | Yes | No | No | No | NR | NR |
| Chung,2015 | South Korea | cohort | 2006-2012 | Bleeding | 465 | 30.1 | 57.1 | 20 | Yes | Yes | No | No | No | NR | Include |
| Das, 2025 | India | cross-sectional | NR | All | 112 | 50.0 | NR | 26 | Yes | Yes | Yes | No | Yes | NR | Exclude |
| Dore, 2019 | Italy | cohort | 2002-2018 | All | 365 | NR | NR | 95 | Yes | No | Yes | No | Yes | NR | Exclude |
| Ebrahimzadeh, 2023 | Iran | cross-sectional | 2005-2012 | Duodenal ulcer | 950 | 38.8 | 47 | 111 | Yes | No | No | No | No | 30 | Include |
| Gisbert1, 1999 | Spain | cohort | NR | Duodenal ulcer | 774 | 30.0 | 46.0 | 6 | Yes | Yes | Yes | No | No | 7 | Include |
| Gisbert2, 2001 | Spain | cohort | NR | Bleeding duodenal ulcer | 92 | 14.0 | 51.0 | 2 | Yes | No | Yes | No | No | 7 | Include |
| Goenka, 2010 | India | cross-sectional | 2008-2009 | All | 192 | 56.3 | 42.3 | 50 | No | Yes | Yes | No | No | NR | NR |
| Golubovic, 2007 | Serbia | cross-sectional | 2003 | Bleeding | 93 | 34.0 | 57.0 | 3 | Yes | No | No | No | No | NR | Include |
| Hasan, 2017 | Bangladesh | cross-sectional | 2014-2015 | All | 138 | NR | NR | 24 | Yes | Yes | Yes | No | No | NR | NR |
| Hikichi, 2018 | Japan | cohort | 2010-2013 | All | 310 | 33.5 | 64.8 | 38 | No | Yes | Yes | Yes | No | NR | Include |
| Hung, 2005 | Hong Kong | cohort | 2000 | Bleeding | 638 | 40.4 | 67.9 | 120 | Yes | Yes | No | No | No | 28 | Include |
| Ishikawa, 2012 | Japan | cohort | 2000-2008 | Bleeding | 305 | 24.9 | 66.7 | 16 | No | No | No | Yes | No | 14 | Include |
| Jang, 2008 | South Korea | cohort | 2004-2005 | All | 895 | 34.5 | 50.7 | 182 | Yes | Yes | No | No | No | 28 | Include |
| Jyotheeswaran, 1998 | USA | cross-sectional | 1993-1996 | All | 305 | NR | NR | 106 | Yes | No | No | No | No | NR | Include |
| Kalaghchi, 2004 | USA | cross-sectional | 1997-1999 | All | 105 | 28.6 | 52.1 | 23 | Yes | No | No | No | No | 28 | NR |
| Kang, 2011 | South Korea | cross-sectional | 2006-2008 | All | 475 | 25.4 | 57.8 | 90 | Yes | Yes | No | Yes | No | 28 | Include |
| Kanno1, 2013 | Japan | cross-sectional | 2012-2013 | All | 382 | 26.2 | 63.2 | 46 | Yes | Yes | Yes | Yes | No | 28 | Include |
| Kanno2, 2015 | Japan | cohort | 2010-2012 | All | 386 | 33.2 | 66.5 | 75 | No | No | No | Yes | No | 28 | Include |
| Kaosombatwattana, 2019 | Thailand | cross-sectional | 2011-2017 | Bleeding | 755 | NR | NR | 117 | Unclear | Unclear | Unclear | Unclear | Unclear | NR | NR |
| Kiatpapan, 2017 | Thailand | cross-sectional | 2003-2013 | All | 1310 | NR | NR | 71 | Yes | Yes | No | No | No | 90 | Include |
| KimJH, 2017 | South Korea | cross-sectional | NR | All | 207 | NR | NR | 29 | No | No | No | Yes | No | NR | NR |
| KimJJ, 2012 | South Korea | cross-sectional | 2007-2008 | All | 310 | 33.3 | 61.5 | 38 | Yes | Yes | No | No | No | NR | Include |
| KimY, 2012 | Japan | cross-sectional | 2004-2009 | Gastric ulcer | 410 | 29.8 | 65.2 | 15 | Yes | Yes | Yes | Yes | No | 7 | Include |
| Konturek, 2003 | Poland | cohort | 1996-2001 | All | 1898 | 48.5 | 49.3 | 355 | No | Yes | Yes | No | No | 56 | Include |
| LeeCC, 2009 | Taiwan | cross-sectional | 2008 | All | 202 | NR | NR | 71 | Yes | Yes | No | No | No | NR | Include |
| LeeIS, 2010 | South Korea | cohort | 2003-2008 | Bleeding | 360 | NR | NR | 42 | Unclear | Unclear | Unclear | Unclear | Unclear | 28 | Include |
| Levenstein, 2017 | Denmark | cohort | 1982-2011 | All | 166 | 50.4 | 53.0 | 47 | No | No | No | Yes | No | 7 | Include |
| Liu, 2008 | Taiwan | cohort | 2005-2006 | Bleeding | 390 | 32.6 | 63.0 | 65 | Yes | Yes | No | Yes | No | 7 | Include |
| Ljubicic, 2012 | Croatia | cohort | 2005-2009 | Bleeding | 1530 | 33.3 | NR | 367 | Yes | No | Yes | No | No | 7 | Include |
| Matsukawa, 2007 | Japan | cross-sectional | NR | Gastric ulcer | 79 | 45.6 | 59.6 | 16 | No | Yes | Yes | No | No | NR | NR |
| Metzger, 2001 | Switzerland | cross-sectional | 1993-1998 | Perforation | 45 | 36.1 | 47.0 | 9 | Yes | No | No | No | No | NR | NR |
| Meucci, 2000 | Italy | cross-sectional | NR | All | 409 | 48.6 | 51.9 | 19 | Yes | Yes | No | No | No | NR | NR |
| Mousavi, 2006 | Italy | cross-sectional | 8 months | Bleeding | 197 | 29.5 | 58.8 | 9 | Yes | Yes | No | Yes | No | 14 | NR |
| Musumba, 2012 | UK | cross-sectional | 2005-2010 | All | 1083 | 46.6 | 64.8 | 59 | Yes | Yes | No | Yes | No | 14 | NR |
| Nensey, 1991 | USA | cross-sectional | 1988-1990 | Duodenum | 52 | 52.7 | 19.2 | 3 | Yes | Yes | No | No | No | NR | Include |
| Ng, 1996 | Hong Kong | cross-sectional | 1996 | Perforation | 73 | 13.7 | 51.0 | 12 | Yes | Yes | No | No | No | NR | NR |
| Nishikawa, 2000 | Japan | cross-sectional | 1992-1997 | All | 398 | 30.2 | 49.7 | 5 | Yes | Yes | Yes | Yes | No | NR | NR |
| Okan, 2003 | Turkey | cross-sectional | 1997-1999 | All | 202 | 32.7 | 52.3 | 12 | No | Yes | No | No | No | 7 | Exclude |
| Ong, 2006 | Singapore | cross-sectional | 2002-2004 | All | 845 | 35.0 | 57.8 | 66 | Yes | Yes | No | No | No | 28 | Include |
| Ootani, 2006 | Japan | cohort | 2002-2004 | Bleeding | 116 | 26.7 | 60.6 | 13 | Yes | Yes | Yes | Yes | No | 28 | Include |
| Pascual, 2003 | Spain | cross-sectional | NR | All | 73 | 19.2 | 63.3 | 1 | No | Yes | No | No | No | NR | NR |
| Pietroiusti, 2008 | Spain | cross-sectional | 2000-2004 | All | 608 | 36.5 | 53.8 | 42 | No | Yes | Yes | No | No | 7 | Include |
| Pillay, 2007 | Malaysia | cross-sectional | 2004-2005 | All | 416 | 30.0 | 56.9 | 108 | No | Yes | No | No | No | NR | NR |
| Publig, 1994 | Austria | cross-sectional | 1992 | All | 82 | 58.5 | 65.1 | 9 | Yes | No | No | Yes | No | 14 | Include |
| Raj, 2001 | Malaysia | cross-sectional | 1996-1997 | All | 124 | 37.9 | NR | 33 | Yes | Yes | No | Yes | No | NR | NR |
| Rajabalinia, 2012 | Iran | cross-sectional | 2010 | Bleeding | 61 | NR | NR | 8 | Unclear | Unclear | Unclear | Unclear | No | NR | NR |
| Ramsoekh, 2005 | Netherlands | cross-sectional | 2000-2001 | Bleeding | 361 | NR | 70.9 | 53 | Yes | No | Yes | No | No | NR | Include |
| Reinbach, 1993 | UK | cross-sectional | NR | Perforation | 80 | 26.0 | 52.0 | 16 | No | No | Yes | Yes | No | NR | NR |
| Rudler, 2011 | France | cross-sectional | 2007-2010 | Bleeding | 188 | NR | NR | 55 | No | No | No | Yes | No | NR | NR |
| Sbrozzi-Vanni, 2010 | Italy | cross-sectional | 2005-2007 | All | 300 | 40.3 | 61.7 | 12 | Yes | No | Yes | No | No | 28 | Include |
| Schilling, 2003 | Germany | cross-sectional | 1996-2000 | Bleeding | 344 | 32.3 | 66.2 | 34 | Yes | Yes | No | No | No | NR | NR |
| Seyed, 2015 | Iran | cross-sectional | 2012-2013 | All | 215 | 37.7 | 62 | 4 | Yes | Yes | No | No | No | 30 | Include |
| Shallcross, 1990 | UK | cross-sectional | NR | All | 70 | NR | NR | 2 | Yes | No | No | No | No | 28 | Exclude |
| Shirvany, 2010 | Iran | cross-sectional | 2004-2008 | All | 749 | NR | 49.3 | 189 | Yes | No | No | No | No | 28 | Include |
| Terai, 2020 | Japan | cohort | 2005, 2018 | Gastric ulcer | 324 | NR | NR | 14 | Yes | No | No | Yes | No | NR | NR |
| Thanapirom, 2015 | Thailand | cross-sectional | NR | Bleeding | 471 | NR | NR | 199 | Unclear | Unclear | Unclear | Unclear | No | NR | NR |
| Thong-Ngam, 2009 | Thailand | cross-sectional | 2004-2005 | All | 30 | 53.3 | 56.2 | 11 | Yes | Yes | No | No | No | 7 | Include |
| Timshina, 2011 | India | cross-sectional | NR | All | 40 | 26.7 | NR | 1 | Yes | Yes | No | No | No | NR | NR |
| Uppalapati, 2009 | USA | cross-sectional | 2004-2005 | All | 230 | NR | NR | 64 | Unclear | Unclear | Unclear | Unclear | Unclear | NR | Include |
| Uyanikoglu, 2012 | Turkey | cross-sectional | 2002-2009 | All | 140 | 42.0 | 47.7 | 17 | Yes | Yes | No | No | Yes | 28 | Include |
| Venerito, 2014 | Germany | cohort | 2002-2004 | All | 1007 | 40.5 | 65.2 | 297 | Yes | Yes | Yes | Yes | No | NR | NR |
| Vestergard, 2009 | Germany | cohort | 2003-2006 | Bleeding | 264 | 48.5 | 72.2 | 17 | Yes | Yes | Yes | No | No | 28 | Include |
| Voutilainen, 2001 | Finland | cross-sectional | NR | All | 76 | 60.8 | 56.0 | 8 | Yes | No | No | No | No | NR | Include |
| Vu, 2000 | Singapore | cross-sectional | 1997 | All | 107 | 43.9 | 65.0 | 12 | Yes | Yes | No | No | No | NR | NR |
| Wyatt, 1992 | UK | cross-sectional | NR | All | 63 | 59.7 | 61.1 | 9 | Yes | No | No | Yes | No | NR | NR |
| XiaB, 2005 | China | cohort | 1997-2003 | All | 386 | 62.2 | 48.7 | 55 | Yes | Yes | Yes | No | No | 90 | NR |
| XiaHH1, 2000 | Australia | cross-sectional | 1996-1998 | All | 45 | 51.6 | 52.1 | 17 | Yes | Yes | No | Yes | No | 90 | Include |
| XiaHH2, 2001 | Hong Kong | Cohort | 1997-1999 | Duodenal ulcer | 599 | 43.1 | 57.9 | 104 | Yes | Yes | Yes | No | No | 90 | Include |
| Yakoob, 2005 | Pakistan | cross-sectional | 1999-2001 | Duodenal ulcer | 217 | 30.4 | 46.4 | 62 | Yes | Yes | No | Yes | No | 28 | Include |
| Yang, 2017 | South Korea | cross-sectional | 2010-2015 | Perforation | 396 | 14.9 | 50.6 | 80 | No | Yes | Yes | Yes | No | NR | Include |
| YoonH, 2013 | South Korea | cross-sectional | 2005 | All | 238 | 34.5 | 59.3 | 56 | Yes | Yes | No | No | No | 28 | Include |
| Zelickson, 2011 | USA | cross-sectional | 2012 | All | 128 | 48.4 | 58.9 | 17 | Yes | Yes | No | Yes | No | NR | NR |
| Zhang, 2003 | China | cross-sectional | 1997-2001 | All | 446 | 17.3 | 46.1 | 99 | Yes | No | No | No | No | 7 | Include |

HP, *H. pylori*; NR, not reported.

**Appendix S6:** Joanna Briggs Institute Critical Appraisal Checklist for Studies Reporting Prevalence Data*

|  | Q1 | Q2 | Q3 | Q4 | Q5 | Q6 | Q7 | Q8 | Q9 | Overall |
| --- | --- | --- | --- | --- | --- | --- | --- | --- | --- | --- |
| Antunes | No | Yes | Yes | Yes | Unclear | Unclear | Unclear | Yes | Unclear | High |
| Aoyama | Unclear | Unclear | Yes | Yes | Unclear | No | Yes | Yes | Unclear | High |
| Archampong | No | Yes | Yes | Yes | Unclear | No | Yes | Yes | Unclear | Moderate |
| Arents | Yes | No | Yes | Yes | Yes | No | Yes | Yes | Yes | Moderate |
| Arroyo | Yes | Yes | Yes | No | No | Yes | Yes | Yes | No | Moderate |
| Auwyang | No | Unclear | Yes | No | Unclear | No | Yes | Yes | Unclear | High |
| Bobrzynski | No | Unclear | Yes | Yes | Unclear | Yes | Yes | Yes | Unclear | Moderate |
| Borody | No | Unclear | Yes | Yes | Unclear | Unclear | Unclear | Yes | Unclear | High |
| Bytzer | Yes | Yes | Yes | Yes | Yes | Yes | Yes | Yes | Yes | Low |
| Carli | No | No | Yes | Yes | Yes | No | Yes | Yes | Yes | Moderate |
| Cekin | Yes | Unclear | Yes | Yes | Unclear | No | Yes | Yes | Unclear | Moderate |
| Chan | No | Yes | Yes | Yes | Yes | No | Yes | Yes | Yes | Moderate |
| Charpignon | Yes | Yes | Yes | Yes | No | Yes | Yes | Yes | No | Moderate |
| Chason | No | Yes | Yes | Yes | No | Yes | Yes | Yes | No | Moderate |
| Chautems | No | Unclear | No | Yes | No | No | Yes | Yes | No | High |
| Chen | No | Unclear | Yes | Yes | Unclear | Yes | Yes | Yes | Unclear | Moderate |
| Chiorean | No | Unclear | Yes | Yes | No | Unclear | Unclear | Yes | No | High |
| Chng | Unclear | Unclear | No | No | Unclear | Yes | Yes | Yes | Unclear | High |
| Choi | Yes | Unclear | Yes | Yes | No | No | Yes | Yes | No | Moderate |
| Chung | Yes | Yes | Yes | Yes | No | No | No | Yes | No | Moderate |
| Das | Yes | Yes | Yes | No | Unclear | No | Yes | Yes | Unclear | Moderate |
| Dore | Yes | No | Yes | Yes | Unclear | Yes | Yes | Yes | Unclear | Moderate |
| Ebrahimzadeh | No | Yes | Yes | Yes | Yes | No | Yes | Yes | Yes | Moderate |
| Gisbert1 | No | Yes | Yes | Yes | Unclear | Yes | Yes | Yes | Unclear | Moderate |
| Gisbert2 | No | Yes | Yes | Yes | Unclear | Yes | Yes | Yes | Unclear | Moderate |
| Goenka | No | Unclear | Yes | Yes | Unclear | No | Yes | Yes | Unclear | High |
| Golubovic | No | Yes | No | Yes | Unclear | No | No | Yes | Unclear | High |
| Hasan | No | Unclear | Yes | Yes | Unclear | Yes | Yes | Yes | Unclear | Moderate |
| Hikichi | Yes | Unclear | Yes | Yes | Unclear | No | Yes | Yes | Unclear | Moderate |
| Hung | Yes | Yes | Yes | Yes | Yes | No | Yes | Yes | Yes | Moderate |
| Ishikawa | No | Yes | Yes | Yes | Unclear | No | Yes | Yes | Unclear | Moderate |
| Jang | Yes | Yes | Yes | Yes | No | No | Yes | Yes | No | Moderate |
| Jyotheeswaran | No | Unclear | Yes | Yes | No | No | Yes | Yes | No | High |
| Kalaghchi | No | Unclear | Yes | Yes | Unclear | No | Yes | Yes | Unclear | High |
| Kang | No | Yes | Yes | Yes | No | Yes | Yes | Yes | No | Moderate |
| Kanno1 | Yes | Yes | Yes | Yes | Yes | Yes | Yes | Yes | Yes | Low |
| Kanno2 | Yes | Yes | Yes | Yes | Yes | No | Yes | Yes | No | Moderate |
| Kaosombatwattana | No | Unclear | Yes | Yes | No | Unclear | Unclear | Yes | No | High |
| Kiatpapan | No | Unclear | Yes | Yes | No | Yes | Yes | Yes | No | Moderate |
| KimJH | No | Yes | Yes | No | Unclear | No | Yes | Yes | Unclear | High |
| KimJJ | No | Unclear | Yes | Yes | No | No | Yes | Yes | No | High |
| KimY | No | Unclear | Yes | Yes | Yes | Yes | Yes | Yes | Yes | Moderate |
| Konturek | No | Yes | Yes | Yes | No | Yes | Yes | Yes | No | Moderate |
| LeeCC | Unclear | Yes | Yes | Yes | Unclear | No | Yes | Yes | Unclear | Moderate |
| LeeIS | No | Yes | Yes | Yes | No | Unclear | Unclear | Yes | No | High |
| Levenstein | Yes | Yes | Yes | Yes | Yes | No | Yes | Yes | Yes | Moderate |
| Liu | No | Yes | Yes | Yes | Yes | Yes | Yes | Yes | Yes | Moderate |
| Ljubicic | Yes | Yes | Yes | Yes | No | Yes | Yes | Yes | No | Moderate |
| Matsukawa | Yes | Unclear | No | Yes | Unclear | Yes | Yes | Yes | Unclear | Moderate |
| Metzger | Yes | Yes | No | Yes | Unclear | No | Yes | Yes | Unclear | Moderate |
| Meucci | Yes | Yes | Yes | No | Unclear | No | Yes | Yes | Unclear | Moderate |
| Mousavi | No | Unclear | Yes | Yes | Unclear | Unclear | Unclear | Yes | Unclear | High |
| Musumba | Yes | Unclear | Yes | Yes | No | Yes | Yes | Yes | No | Moderate |
| Nensey | No | Yes | No | Yes | Yes | No | Yes | Yes | Yes | Moderate |
| Ng | No | Yes | Yes | Yes | Unclear | Yes | Yes | Yes | Unclear | Moderate |
| Nishikawa | No | Unclear | Yes | Yes | Unclear | Yes | Yes | Yes | Unclear | Moderate |
| Okan | No | Unclear | Yes | Yes | No | No | Yes | Yes | No | High |
| Ong | Yes | Yes | Yes | Yes | No | No | Yes | Yes | No | Moderate |
| Ootani | No | No | Yes | Yes | Unclear | Yes | Yes | Yes | Unclear | Moderate |
| Pascual | No | Yes | No | No | No | No | Yes | Yes | No | High |
| Pietroiusti | No | Yes | Yes | Yes | No | Yes | Yes | Yes | No | Moderate |
| Pillay | No | Yes | Yes | Yes | Unclear | No | Yes | Yes | Unclear | Moderate |
| Publig | No | Yes | No | Yes | Unclear | No | Yes | Yes | Unclear | High |
| Raj | No | Yes | No | Yes | Unclear | Yes | Yes | Yes | Unclear | Moderate |
| Rajabalinia | No | No | No | Yes | Unclear | Unclear | Unclear | Yes | Unclear | High |
| Ramsoekh | Yes | Yes | Yes | Yes | No | Yes | Yes | Yes | No | Moderate |
| Reinbach | Yes | Yes | Yes | No | Unclear | No | Yes | Yes | Unclear | Moderate |
| Rudler | No | Unclear | Yes | Yes | Unclear | No | Yes | Yes | Unclear | High |
| Sbrozzi-Vanni | Yes | Yes | Yes | Yes | Yes | Yes | Yes | Yes | Yes | Low |
| Schilling | No | Yes | Yes | Yes | Unclear | No | Unclear | Yes | Unclear | High |
| Seyed | No | Yes | Yes | Yes | Unclear | No | Yes | Yes | Unclear | Moderate |
| Shallcross | No | Yes | No | No | Yes | No | Yes | Yes | Yes | Moderate |
| Shirvany | No | No | Yes | Yes | No | No | Yes | Yes | No | High |
| Terai | Yes | Unclear | Yes | Yes | Unclear | Yes | Yes | Yes | Unclear | Moderate |
| Thanapirom | Yes | Unclear | Yes | Yes | Unclear | Unclear | Unclear | Yes | Unclear | High |
| Thong-Ngam | No | Unclear | No | Yes | No | No | Yes | Yes | No | High |
| Timshina | No | Yes | No | No | Unclear | No | Yes | Yes | Unclear | High |
| Uppalapati | Yes | Unclear | Yes | Yes | No | Yes | Yes | Yes | No | Moderate |
| Uyanikoglu | No | Yes | Yes | Yes | Unclear | Yes | Yes | Yes | Unclear | Moderate |
| Venerito | No | Unclear | Yes | Yes | Unclear | Yes | Yes | Yes | Unclear | Moderate |
| Vestergard | No | Yes | Yes | Yes | No | Yes | Yes | Yes | No | Moderate |
| Voutilainen | Yes | Yes | No | No | Yes | No | Yes | Yes | Yes | Moderate |
| Vu | No | Yes | Yes | Yes | Unclear | No | Yes | Yes | Unclear | Moderate |
| Wyatt | No | Yes | No | No | No | Yes | Yes | Yes | No | High |
| XiaB | Yes | Yes | Yes | Yes | Yes | Yes | Yes | Yes | Yes | Low |
| XiaHH1 | No | Yes | No | Yes | Yes | Yes | Yes | Yes | Yes | Moderate |
| XiaHH2 | No | Yes | Yes | Yes | No | Yes | Yes | Yes | No | Moderate |
| Yakoob | No | Unclear | Yes | Yes | Yes | No | Yes | Yes | Yes | Moderate |
| Yang | Yes | Unclear | Yes | Yes | No | Yes | Yes | Yes | No | Moderate |
| YoonH | No | Unclear | Yes | Yes | No | Yes | Yes | Yes | No | Moderate |
| Zelickson | No | No | Yes | Yes | Unclear | Yes | Yes | Yes | Unclear | Moderate |
| Zhang | No | Unclear | Yes | Yes | Unclear | No | Yes | Yes | Unclear | High |

Q1. Was the sample frame appropriate to address the target population?

Q2. Were study participants sampled in an appropriate way?

Q3. Was the sample size adequate?

Q4. Were the study subjects and the setting described in detail?

Q5. Was the data analysis conducted with sufficient coverage of the identified sample?

Q6. Were valid methods used for the identification of the condition?

Q7. Was the condition measured in a standard, reliable way for all participants?

Q8. Was there appropriate statistical analysis?

Q9. Was the response rate adequate, and if not, was the low response rate managed appropriately?

*Risk of bias was rated on nine domains using the Joanna Briggs Institute Critical Appraisal Checklist for Studies Reporting Prevalence Data by two reviewers. As the JBI tool does not generally recommend deriving a single overall risk-of-bias judgment, we adapted the tool for the purposes of this review and applied prespecified decision rules to assign an overall study-level rating. Studies with all items rated Yes will be considered low risk of bias, those with up to four No and/or Unclear ratings will be considered moderate risk of bias, and those with five or more No and/or Unclear ratings will be considered high risk of bias. Disagreements will be resolved by discussion, with a third reviewer acting as arbiter if required.

**Appendix S7.** Subgroup analyses of non-H. pylori, non-NSAID peptic ulcer disease proportion among gastric peptic ulcer disease

| Variable | Subgroup | No. of studies | Pooled proportion, % | 95% CI, % | 95% PI, % | P value^*^ |
| --- | --- | --- | --- | --- | --- | --- |
| Study period duration | ≤1 year | 10 | 16.5 | 10.0–25.8 | 3.3–53.3 | 0.72 |
|  | 1–3 years | 12 | 15.6 | 9.1–25.4 | 2.2–60.5 |  |
|  | 3–5 years | 5 | 11.1 | 3.3–31.2 | 0.5–73.8 |  |
|  | >5 years | 3 | 9.3 | 5.1–16.4 | 3.0–25.4 |  |
| Study design | Cohort | 7 | 13.5 | 8.5–20.7 | 3.7–38.9 | 0.93 |
|  | Cross-sectional | 28 | 13.7 | 9.5–19.4 | 1.9–56.4 |  |
| Geographic region | Asia-Pacific | 20 | 16.2 | 11.0–23.4 | 2.5–59.2 | 0.09 |
|  | Europe | 10 | 7.5 | 4.6–11.9 | 1.9–24.7 |  |
|  | Middle East | 1 | 36.4 | 31.1–42.0 | – |  |
|  | North America | 3 | 18.1 | 6.3–42.1 | 2.1–69.3 |  |
|  | Other regions | 1 | 10.8 | 5.7–19.5 | – |  |
| Decade of study | 1990s | 10 | 11.4 | 5.7–21.7 | 1.2–58.3 | 0.64 |
|  | 2000s | 19 | 15.6 | 10.3–22.8 | 2.5–57.4 |  |
|  | 2010s | 6 | 11.9 | 7.4–18.7 | 3.4–34.0 |  |

*P values are for between-subgroup differences.

CI, confidence interval; PI, prediction interval.

For gastric ulcer, the pooled proportion of non–H. pylori, non-NSAID ulcer was 13.7% (35 studies; 95% CI, 10.1%–18.3%; I² = 96.9%). No statistically significant subgroup differences were identified. In site-specific meta-regression, study year was not statistically significantly associated with the pooled proportion (35 studies; β = 0.028; 95% CI, －0.026 to 0.082; p = 0.31).

**Appendix S8.** Subgroup analyses of non-H. pylori, non-NSAID peptic ulcer disease proportion among duodenal peptic ulcer disease

| Variable | Subgroup | No. of studies | Pooled proportion, % | 95% CI, % | 95% PI, % | P value^*^ |
| --- | --- | --- | --- | --- | --- | --- |
| Study period duration | ≤1 year | 10 | 12.6 | 7.5–20.6 | 2.4–46.4 | 0.39 |
|  | 1–3 years | 16 | 14.0 | 9.2–20.7 | 2.6–50.1 |  |
|  | 3–5 years | 4 | 6.8 | 2.4–17.5 | 0.8–40.5 |  |
|  | >5 years | 4 | 7.8 | 3.3–17.5 | 1.1–38.3 |  |
| Study design | Cohort | 6 | 13.1 | 7.0–23.1 | 2.5–46.8 | 0.42 |
|  | Cross-sectional | 34 | 9.1 | 6.3–12.9 | 1.1–47.0 |  |
| Geographic region | Asia-Pacific | 20 | 14.3 | 9.9–20.2 | 2.7–49.9 | 0.028 |
|  | Europe | 14 | 4.9 | 2.8–8.2 | 0.7–26.5 |  |
|  | Middle East | 1 | 11.7 | 9.7–13.9 | – |  |
|  | North America | 4 | 12.9 | 4.1–33.9 | 1.0–68.2 |  |
|  | Other regions | 1 | 6.9 | 2.6–14.4 | – |  |
| Decade of study | 1990s | 14 | 7.1 | 3.4–14.3 | 0.4–56.2 | 0.54 |
|  | 2000s | 19 | 11.1 | 7.4–16.4 | 1.8–45.4 |  |
|  | 2010s | 6 | 13.5 | 8.6–20.6 | 4.4–34.5 |  |

*P values are for between-subgroup differences.

CI, confidence interval; PI, prediction interval.

For duodenal ulcer, the pooled proportion of non–H. pylori, non-NSAID ulcer was 9.7% (40 studies; 95% CI, 7.0%–13.2%; I. = 97.3%). Geographic subgroup differences were observed (p = 0.028), with higher pooled proportions in Asia-Pacific (14.3%) and lower pooled proportions in Europe (4.9%). In site-specific meta-regression, a later study year was associated with a higher pooled proportion (40 studies; β = 0.057; 95% CI, 0.006 to 0.107; p = 0.03).

**Appendix S9.** Sensitivity analyses of study in univariable meta-regression analyses of study-level factors associated with the proportion of non-*H. pylori*, non-NSAID peptic ulcer disease in bleeding peptic ulcer disease cases

| Clinical presentation | Moderator^†^ | k | p-value | Estimates (95% CI)^*^ |
| --- | --- | --- | --- | --- |
| Main analysis (random-effect) | Study year, year | 35 | <0.001 | 0.076 (0.03 to 0.12) |
| Published studies only (random-effect) | Study year, year | 28 | 0.02 | 0.061 (0.008 to 0.11) |
| Known etiology only (random-effect) | Study year, year | 35 | 0.003 | 0.06 (0.008 to 0.113) |
| Studies using ≥2 diagnostic methods for *H. pylori* | Study year, year | 22 | 0.12 | 0.056 (−0.01 to 0.13) |
| Studies with a clearly defined NSAID exposure window | Study year, year | 21 | 0.003 | 0.094 (0.033 to 0.156) |
| Studies explicitly classifying aspirin as an NSAID | Study year, year | 24 | 0.031 | 0.072 (0.006 to 0.138) |
| Studies with a clearly defined study period | Study year, year | 33 | <0.001 | 0.074 (0.031 to 0.117) |
| All four definitional criteria met^‡^ | Study year, year | 13 | 0.066 | 0.078 (−0.005 to 0.162) |

^*^Estimates represent regression coefficients from univariable random-effects meta-regression models on the logit scale

^†^Study year is defined as the midpoint of the reported study period. Study period duration is the length of the study period in years

^‡^Defined as studies that met all four criteria: ≥2 H. pylori diagnostic methods, aspirin classified as an NSAID, clear NSAID exposure window, and clear study period.

CI, confidence interval

**Appendix S10.** Sensitivity analyses of non-H. pylori, non-NSAID peptic ulcer disease proportion among bleeding peptic ulcer disease

| Variable | No. of studies | Pooled proportion % | 95% CI, % | 95% PI, % | I^2^, % |
| --- | --- | --- | --- | --- | --- |
| Main analysis (random-effect) | 35 | 11.3 | 8.8–14.4 | 2.6–38.0 | 95.5 |
| Main analysis (fixed-effect) | 35 | 17.4 | 16.6–18.2 | – | 94.7 |
| Published studies only (random-effect) | 28 | 10.0 | 7.7–13.0 | 2.5–32.5 | 94.2 |
| Known etiology only (random-effect) | 35 | 12.2 | 9.6–15.5 | 2.9–39.3 | 95.2 |
| Studies using ≥2 diagnostic methods for *H. pylori* | 22 | 11.5 | 8.5–15.4 | 2.8–37.2 | 95.1 |
| Studies with a clearly defined NSAID exposure window | 21 | 10.0 | 7.3–13.6 | 2.4–34.0 | 95.0 |
| Studies explicitly classifying aspirin as an NSAID | 24 | 11.0 | 8.1–14.9 | 2.3–39.0 | 95.8 |
| Studies with a clearly defined study period | 33 | 11.7 | 9.1–14.8 | 2.9–37.1 | 95.3 |

*P values are for between-subgroup differences.

CI confidence interval; PI, prediction interval.

**Appendix S11.** Subgroup analyses of non-H. pylori, non-NSAID peptic ulcer disease proportion among bleeding gastric peptic ulcer disease

| Variable | Subgroup | No. of studies | Pooled proportion, % | 95% CI, % | 95% PI, % | P value^*^ |
| --- | --- | --- | --- | --- | --- | --- |
| Study period duration | ≤1 year | 5 | 10.7 | 4.1–24.9 | 1.1–56.0 | 0.49 |
|  | 1–3 years | 2 | 14.7 | 10.6–19.9 | 10.4–20.3 |  |
|  | >5 years | 3 | 6.7 | 5.0–9.0 | 5.0–9.0 |  |
| Study design | Cohort | 1 | 25.4 | 15.9–38.0 | – | 0.13 |
|  | Cross-sectional | 9 | 9.0 | 5.6–14.1 | 2.2–30.3 |  |
| Geographic region | Asia-Pacific | 7 | 9.0 | 5.0–15.7 | 1.8–35.1 | 0.67 |
|  | Europe | 2 | 8.8 | 1.5–37.3 | 0.5–63.4 |  |
|  | North America | 1 | 18.2 | 14.1–23.2 | – |  |
| Decade of study | 1990s | 2 | 4.5 | 1.6–11.6 | 0.8–20.5 | 0.37 |
|  | 2000s | 8 | 12.6 | 8.2–18.7 | 3.9–33.5 |  |

*P values are for between-subgroup differences.

CI confidence interval; PI, prediction interval.

For bleeding gastric ulcer, the pooled proportion of non–H. pylori, non-NSAID ulcer was 10.0% (10 studies; 95% CI, 6.2%–15.7%; I. = 90.7%). No statistically significant subgroup differences were identified. Site-specific meta-regression did not show a statistically significant association with study year.

**Appendix S12.** Subgroup analyses of non-H. pylori, non-NSAID peptic ulcer disease proportion among bleeding duodenal peptic ulcer disease

| Variable | Subgroup | No. of studies | Pooled proportion, % | 95% CI, % | 95% PI, % | P value^*^ |
| --- | --- | --- | --- | --- | --- | --- |
| Study period duration | ≤1 year | 6 | 10.6 | 5.7–18.7 | 2.5–35.2 | 0.45 |
|  | 1–3 years | 3 | 17.0 | 9.0–29.7 | 5.3–42.5 |  |
|  | >5 years | 4 | 11.7 | 9.0–15.2 | 9.0–15.2 |  |
| Study design | Cohort | 1 | 16.7 | 7.7–32.5 | – | 0.52 |
|  | Cross-sectional | 13 | 10.7 | 7.4–15.3 | 3.2–30.4 |  |
| Geographic region | Asia-Pacific | 10 | 12.3 | 8.2–18.1 | 3.8–33.2 | 0.36 |
|  | Europe | 3 | 5.5 | 1.6–17.0 | 0.6–35.8 |  |
|  | North America | 1 | 13.6 | 9.5–19.1 | – |  |
| Decade of study | 1990s | 4 | 8.8 | 2.9–23.4 | 0.8–53.2 | 0.61 |
|  | 2000s | 10 | 13.0 | 10.5–16.1 | 8.7–18.9 |  |

*P values are for between-subgroup differences.

CI, confidence interval; PI, prediction interval.

For bleeding duodenal ulcer, the pooled proportion of non–H. pylori, non-NSAID ulcer was 11.1% (14 studies; 95% CI, 7.9%–15.5%; I. = 81.5%). No statistically significant subgroup differences were identified. Site-specific meta-regression did not show a statistically significant association with study year.
